# Evaluation of minimally invasive metabolomic methods for assessing the health of sturgeons

**DOI:** 10.1038/s41598-025-17654-2

**Published:** 2025-10-02

**Authors:** Timothy W. Collette, Shannon N. Romano, Quincy Teng, Adam G. Fox, J. Shane Kornberg, Drew R. Ekman

**Affiliations:** 1 Center for Environmental Measurement and Modeling, U.S. EPA, Athens, GA USA; 2 Warnell School of Forestry and Natural Resources, University of Georgia, Athens, GA USA

**Keywords:** Environmental sciences, Metabolomics

## Abstract

Almost all species of sturgeon are at risk of extinction throughout their natural range due to a variety of human activities. As a result, there is now a global effort focused on their preservation. For maximum success, sturgeon restoration and reintroduction activities should incorporate information-rich methods for monitoring the health and well-being of sturgeon populations. However, only non-lethal, minimally invasive techniques should be employed due to their status as endangered species. Metabolomics-based monitoring using blood and epidermal mucus has shown considerable potential for application to sensitive or endangered species such as sturgeons. Here, we report the nuclear magnetic resonance (NMR)-detectable serum and epidermal mucous metabolomes of Russian and Siberian sturgeon, with extensive endogenous metabolite assignments. Serum samples were further characterized for higher molecular weight components (e.g., lipoproteins and acute phase proteins) that are closely associated with growth, development, and stress response. Multi- and univariate statistical analyses of these datasets suggest that the Russian sturgeon blood serum metabolome and the Siberian sturgeon epidermal mucous metabolome are notably sex specific. Thus, these datasets provided rich biochemical information useful for improving assessments of both general and sex-specific health, including impacts from environmental stressors (chemical contaminants, temperature extremes, etc.), for wild sturgeon populations.

**Supplementary Information:**

The online version contains supplementary material available at 10.1038/s41598-025-17654-2.

## Introduction

The approximately 25 extant species of fish commonly known as sturgeon, belonging to the family *Acipenseridae*, are among the oldest bony fishes in existence. Native within a large expanse of the temperate northern hemisphere, sturgeon species are generally quite large (often > 45 kg) and long-lived (lifespan in some species exceeds 100 years). However, despite their impressive size and longevity, they are also among the most imperiled fishes in the world^1^; a result of anthropogenic effects including overharvest, pollution, and dams that block spawning migrations. Indeed, most sturgeon species are currently classified as endangered or critically endangered^2^. As a result, there is now considerable research, restoration, and reintroduction activity focused on the preservation of sturgeon around the globe, both for the goal of conservation and to establish or maintain viable fisheries^3^.

Success in these global restoration activities will require information-rich methods for monitoring the health and well-being of both indigenous and reintroduced sturgeon populations. For example, it will be important to monitor the growth and development of animals released as juveniles, and to ascertain their health and reproductive viability as they develop into adulthood. However, due to their endangered status, and to the laws and regulations that protect such species in many countries; only non-lethal and, moreover, minimally invasive methods can be employed. Metabolomics, a relatively new technique in the ‘omics toolbox that involves the profiling of all measurable endogenous metabolites in an organism’s tissues or biofluids, has shown particular promise for application to sensitive or endangered species. This is due not only to the physiological relevance of the measured endpoints, but also because multiple biofluids rich with endogenous metabolites can often be collected with minimal impact on the organism^4^. For fish, this primarily includes blood^5^ urine^6^ and epidermal mucus^7^. Although applications of metabolomics in fish biochemistry and physiology employing these biofluids have typically been directed at characterizing responses to external stressors (e.g., chemical pollutants, etc.)^8^ the metabolome can also serve as an effective indicator of health due to the abundance of biochemical information that is captured^9^. Indeed, metabolomics has now matured to the point of being utilized, for example, in infectious fish disease management and a variety of other fish disease studies^10^.

Interestingly, metabolomic studies with fish have often also revealed clear differences in metabolite profiles when comparing adult males and females^11^. This includes differences in unstressed (i.e., control) fish that are frequently larger than the perturbations associated with sublethal stress responses, e.g., due to exposure to trace levels of chemical contaminants^7^. Thus, metabolomics also offers the potential for sex-specific health monitoring for improving assessments of wild populations. This is a critical aspect of fisheries management given the well-recognized disparities between the sexes regarding stress, the immune response, bioenergetics, and various other physiological parameters that are known to affect fish health^12^. Note that sex differences in metabolomes have been reported (for example, with *Pimephales promelas* (fathead minnow)) not only for sexually dimorphic organs like liver^11^ but also for epidermal mucus^4^. Thus, the potential for sex-specific monitoring of other fish species using the epidermal mucous metabolome—which can be collected non-invasively—seems plausible.

For the current study, we collected both epidermal mucus and blood serum from a population of age-identified adult male and female Russian sturgeon (*Acipenser gueldenstaedtii*) and adult male and female Siberian sturgeon (*Acipenser baerii*), which were captive at a facility that was engaged in caviar production. Endogenous metabolite profiles of these mucus and serum samples were measured by^1^H NMR spectroscopy and extensively characterized. These data allowed us not only to assess the potential for metabolite profiles collected non-lethally to serve as indicators of overall sturgeon health, but also to evaluate the sex specificity of these profiles to provide additional value to health and population assessments. Additionally, we conducted cross-species comparisons of blood serum and epidermal mucus profiles to further inform the development of nonlethal metabolomics tools for sturgeon monitoring.

## Materials and methods

### Sample collection

Sexed and aged adult Russian and Siberian sturgeon were housed and sampled during July 2021 at the Cohutta Fisheries Center, Cohutta, GA, an extension facility of the University of Georgia’s Warnell School of Forestry and Natural Resources. Prior to sampling, roughly equal numbers of males and females (sex previously determined using laparoscopy) of a given species were held in separate holding tanks. Each fish (one-by-one) was manually removed from their holding tank, weighed, measured, sampled for blood and mucus, and then returned to their tank within approximately five minutes, with the goal of minimizing impacts of sampling stress. (Note that no fish displayed overt signs of stress once returned to their tanks.) The number (N) of fish thus sampled was: nine male Russian sturgeon that were hatched in 2011 (i.e., were from the 2011 cohort and were, therefore, 10 years old at the time of sampling), 10 female Russian sturgeon (2011 cohort, 10 years old), five male Siberian sturgeon (2003 cohort, 18 years old), and five female Siberian sturgeon (2006 cohort, 15 years old). Note that these fish were all hatched onsite at the Cohutta facility from eggs that were acquired from AquaTech (Kitzbühel, Austria, Europe).

Whole blood was collected from the caudal vein and dispensed into serum separator tubes. After a 30-min incubation at room temperature, serum was isolated from the clotted whole blood by centrifugation at 3000 g for 15 min, aliquoted into microcentrifuge tubes and transported on dry ice to the U.S. Environmental Protection Agency (EPA) laboratory in Athens, GA. Serum samples were then stored at − 80 °C until processed for analyses.

Epidermal mucus was collected from the ventral side of each fish by placing, and then gently patting, a round 90 mm glass-fiber filter paper disk (GE Bio-sciences 1820-090) between the pelvic fin and the anal fin. Each disk was then inserted into a 15 mL conical tube and placed on dry ice for transportation to the EPA-Athens lab. The disks were then stored at − 80 °C until the epidermal mucus was processed for analysis.

All experimental protocols were approved by the University of Georgia Institutional Animal Care and Use Committee, and all methods were carried out in accordance with relevant guidelines and regulations. The study is reported in accordance with ARRIVE guidelines.

### Sample preparation and data collection

Blood serum samples were thawed on ice; 65 µL of serum was combined with 135 µL of 0.1 M sodium phosphate buffered deuterium oxide (pH 7.4) containing 20 µM sodium 2,2-dimethyl-2-silapentane-5-sulfonate-d_6_ (DSS-d_6_) as an NMR chemical shift reference and transferred to a 3 mm NMR tube.

To extract mucus samples from the glass filter fiber paper disks, each disk was thawed on ice and a one-quarter section of the paper was removed for processing. Each one-quarter section was cut into four strips that were placed in two consecutive wells (two strips per well) of a 96-well protein precipitation filter plate (0.2 μm filter pore; Agilent A5969002), which was then placed on top of a 1-mL 96-well collection plate (Wheaton Microliter 07–6017 N). The filter paper strips were then soaked in methanol for 10 min and filtered by centrifugation for 10 min at 3000 g at 4 °C. After the filtered eluate was collected, the pair of eluates corresponding to a given fish were combined into a single sample. Each sample was then extracted using a published dual-phase process^13^ after which polar and non-polar extracts were dried under vacuum (only results from the polar extracts are reported herein). The dried polar extracts were reconstituted in 70 µL of 0.1 M sodium phosphate buffered deuterium oxide (pH 7.4) containing 20 µM DSS-d_6_ and transferred to a 1.7 mm NMR tube.

One-dimensional (1D) ^1^H NMR data were acquired at 20 °C using a Bruker Avance 600 MHz NMR spectrometer (Bruker, Billerica, MA) equipped with a 5 mm cryoprobe and SampleJet autosampler. Blood serum spectra were collected using the Carr-Purcell-Meiboom-Gill (CPMG) pulse sequence with 128 transients (i.e., scans) per spectrum^14^. Epidermal mucus spectra were collected using the 1D ^1^H Nuclear Overhauser Effect Spectroscopy (NOESY) pulse sequence with a 10-ms mixing time and 1024 transients. Both pulse sequences employed a 2-s pre-saturation pulse to suppress the residual water signal and a 2-s acquisition time.

A set of two-dimensional (2D) NMR spectra were collected with 32 transients and 2048 free induction decays (FIDs) at 20 °C using a Bruker 800 MHz NMR spectrometer equipped with a 1.7 mm cryoprobe. The 2D spectra were used solely to aid in endogenous metabolite resonance assignments. Metabolite resonance assignments were also aided by use of both the Chenomx NMR Suite 8.6 (Chenomx Inc., Edmonton, AB, Canada) and previously published values^15^.

Blood serum contains both low molecular weight (< 1000 Da) metabolites that are central to metabolic pathways (e.g., glucose), and relatively higher molecular weight (> 1000 Da) proteins that, for example, transport lipids (e.g., lipoproteins) and respond to stress (e.g., acute phase proteins). Diffusion-edited NMR spectroscopy, which selects for these larger molecular weight components (that diffuse more slowly in solution), was used to identify peaks in the spectra that belong to lipoproteins and acute phase proteins. Diffusion-edited spectra were collected using the ledbpgppr2s1d pulse sequence with a 120-ms diffusion delay, 100% gradient power and a 2.2-s acquisition time. A 2-s pre-saturation pulse was used to suppress the residual water signal. Peak identities were assigned using previously published values^16^.

### Data processing and analysis

1D ^1^H NMR data were processed with the ACD/Spectrus Processor 2020.1.0 (ACD Labs, Toronto, Ontario, Canada) using 0.3 Hz line broadening followed by automated phase and baseline correction. Spectra were then imported into SpecAlign^17^ for chemical-shift alignment and subsequently imported back to ACD/Spectrus where they were segmented into 0.005-ppm bins within the range of 0.50–10.00 ppm. The binned spectra were then exported to Excel (Microsoft Corporation, Redmond, WA, U.S.A.) where peaks from the residual water resonance (4.70–5.10 ppm) were removed from all spectra. Resonances from acetate, methanol, and formate (1.90–1.93 ppm, 3.35–3.36 ppm, and 8.40–8.50 ppm, respectively) were also removed from the epidermal mucus spectra due to their occurrence in associated method blanks. All spectra were then normalized to unit total intensity. Thus, after normalization, a given peak abundance did not reflect its absolute metabolite concentration; instead, it reflected its concentration relative to all other detected metabolites.

The binned and normalized spectra were imported into SIMCA-17.0 (Sartorius, Göttingen, Germany) and assessed using principal component analysis (PCA). First, outlier analysis was conducted with PCA using the Hotelling’s *T*^2^ test at the 95% confidence interval; only one sample (epidermal mucus from a female Siberian) was excluded as an outlier. PCA score plots from unscaled, mean-centered data were then used to visualize relative differences in the metabolomes of males and females within each species, as well as the differences in the metabolomes between species. The p-values that describe comparisons of PCA score values among these sexes and species were determined using two-tailed Students t-tests; normality was determined using the Kolmogorov–Smirnov test, and Levene’s test was used to determine the equality of variance.

The binned and normalized spectra were also used in Excel to construct “*t*-test filtered difference spectra,” which aided the identification of metabolites that differed significantly (*p* < 0.05) with regard to sex, and also those metabolites that differed with regard to species. Details for the approach to constructing t-test filtered difference spectra, including the procedure for controlling for false positives, have been described previously^18^. To further evaluate impacts of sex and species on metabolite composition and abundance, we calculated the total intensity values for these t-test filtered difference spectra by summing the absolute values of the magnitudes for all significantly different bins. This approach provided a single and easily visualized metric that integrated the breadth and magnitude of endogenous metabolite differences across sex or species^8^. A one-way ANOVA with posthoc Tukey’s test was used to assess whether these total intensity values (for example, when comparing sexes) differed (at *p* < 0.05) among the various species and sample types.

### Comparison to a sexually dimorphic fish

Note that we had previously collected samples of adult fathead minnow blood serum and epidermal mucus for other purposes^4^; a number of these samples were processed and analyzed by NMR in a similar fashion, and the resultant data was handled in the same manner, as described above for the sturgeon samples. Specifically, the number of fathead minnow samples thus processed was eight male and eight female blood serum samples, and 12 male and 12 female epidermal mucus samples. In addition, we used raw data reported in an earlier publication to generate—using the same data handling approaches as described above for the sturgeon—the total intensity value for sex specificity in the fathead minnow hepatic metabolome (polar-extract, *N* = 8 males and 8 females)^19^.

## Results

### Blood serum metabolome characterization for Russian and Siberian sturgeon

The low molecular weight endogenous metabolites identified by NMR in blood serum for Russian sturgeon and Siberian sturgeon are tabulated in the supporting information (Supplementary Tables S1 and S2, respectively). Additionally, representative NMR spectra are presented in Supplementary Figs. S1 and S2; these reflect the relative intensities of metabolites and proteins for each sex and both species. (Note that the key that associates the spectrum peak-label numbers in Figs. S1 and S2 with the metabolites or proteins that give rise to the peaks is in Supplementary Table S3.) Numerous metabolite classes were detected including carbohydrates (e.g., glucose), amino acids (e.g., glutamine), organic acids (e.g., citrate), and amines (e.g., betaine). Higher molecular weight components (e.g., lipoproteins, acute phase proteins (GlycA)) were also detected as confirmed by collecting 1D diffusion-edited spectra (Supplementary Fig. S3).

**Fig. 1:**
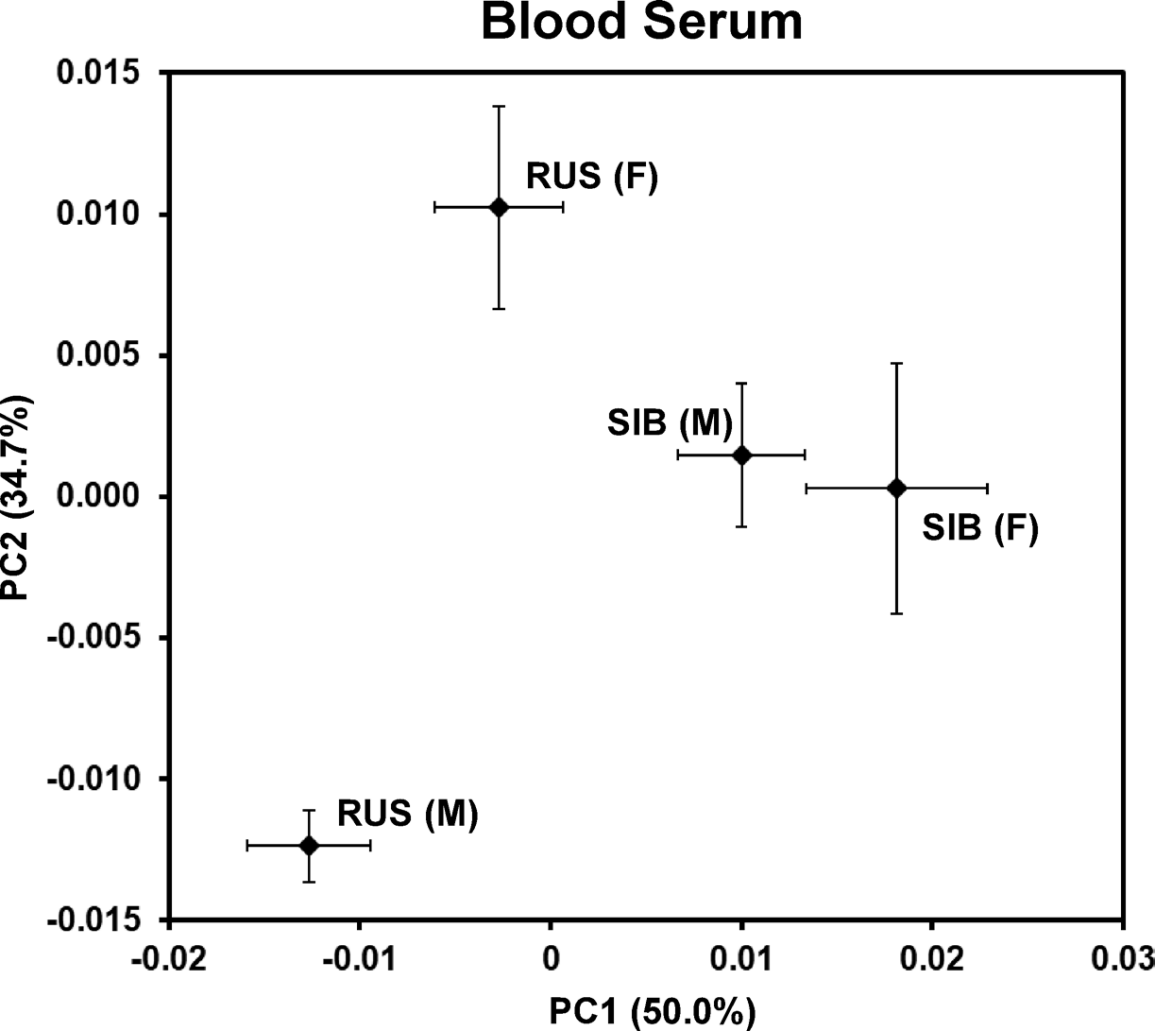
Score plot from principal components analysis (PCA) that compared blood serum ^1^H NMR spectra from each sampled sturgeon species and sex. Markers are mean score values for the first two principal components (PC1 and PC2). Error bars represent standard errors of the means. The percent of variance in the dataset captured by each component is shown parenthetically in the axis titles. RUS, Russian sturgeon; SIB, Siberian sturgeon; M, male; F, females.

A score plot from the PCA model that compared NMR-detectable endogenous metabolites in blood serum from the male and female Russian and Siberian sturgeon is shown in Fig. 1. Score values for the first two principal components (PC1 and PC2) are displayed as the mean (± standard error) for all sampled fish of a given sex and species. It is important to note that a separation in score values in the X direction (along PC1) depicts a greater difference in the metabolomes than does a separation of the same extent along PC2, because, by definition, PC1 captures the most variability in the data. Thus, in Fig. 1, the greatest difference in metabolomes is observed when comparing Russian sturgeon with Siberian sturgeon. Indeed, scores for both males and females are significantly different along PC1 when comparing across these species (*p* = 0.0007 and 0.0339, respectively). Furthermore, the serum metabolomes of male and female Russian sturgeon differ to a much greater extent (*p* = 9.2E−05 along PC2) than do the metabolomes of male and female Siberian sturgeon (*p* = 0.8222 along PC2).

**Fig. 2:**
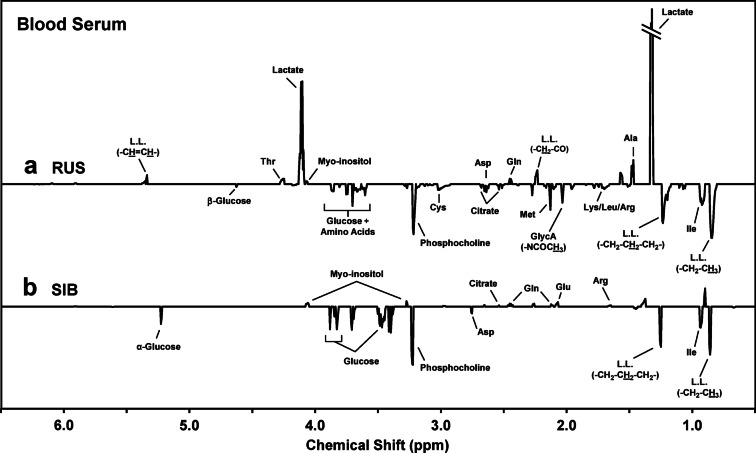
Average ^1^H NMR difference spectra, over the region 6.5–0.5 ppm, that compare the relative abundances of endogenous metabolites measured in the serum of: (**a**) male versus female Russian sturgeon, and (**b**) male versus female Siberian sturgeon. Positive-going peaks represent metabolites that are relatively more abundant in females, while negative-going peaks represent those that are relatively more abundant in males. Only those peaks for which differences were determined to be significant (Student’s *t*-test, *p* < 0.05, see “Materials and methods” section for details) were included. Note that the two difference spectra are displayed using the same Y-axis scale, and that the positive-going peak for lactate (1.33 ppm) in the top spectrum is off scale. Also, note that the hydrogens (H) that are underlined in certain peak labels are the atoms responsible for the labeled peak. These spectra are shown over the full range of 9.5–0.5 ppm in Supplementary Fig. S4. RUS, Russian sturgeon; SIB, Siberian sturgeon; Ala, alanine; Arg, arginine; Asp, aspartate; Cys, cysteine; GlycA, glycosylated acute phase protein A; Gln, glutamine; Ile, isoleucine; Leu, leucine; L.L., lipoprotein lipids; Lys, lysine; Met, methionine; Thr, threonine.

To determine the metabolites that contributed to the observed differences in sexes for a given species, we constructed the t-test filtered difference spectra displayed in Fig. 2. Note that these spectra were generated by subtracting the average spectrum for males of a given species from that of the females. Thus, positive-going peaks represent metabolites that are relatively more abundant (with statistical significance, *p* < 0.05) in females, while negative-going peaks represent those that are relatively more abundant in males. Also, note that the height of a peak reflects the extent to which the associated metabolite abundance differs across sexes. Thus, for the case of Russian sturgeon (Fig. 2a), the most notable difference in metabolite profiles is the greater abundance in lactate for females as compared to males (peaks at 1.33 and 4.11 ppm). To a lesser extent, we also observe a greater abundance of alanine and several lipoprotein-based lipids in female Russian sturgeon. Among the metabolites found to be in greater abundance in male Russian sturgeon (as compared to the females) are citrate, phosphocholine, and glucose. We also observed sex-based differences in some lipoproteins with the male Russian sturgeon displaying greater abundances.

The *t*-test filtered difference spectrum that compares male and female Siberian sturgeon metabolomes (Fig. 2b) is displayed using the same Y-axis scale as for the analogous spectrum for Russian sturgeon (Fig. 2a). Therefore, it is clear when comparing these two spectra that no metabolite differences among male and female Siberian sturgeon rival the magnitude of the sex-based difference observed for lactate in the Russian sturgeon serum metabolomes. However, there were a considerable number of metabolites whose abundances were significantly different between the sexes for Siberian sturgeon. For example, as with the Russian sturgeon, some lipoproteins were observed at higher levels in males. Glucose also displayed this bias. Conversely, the amino acids glutamine and glutamate, and the sugar alcohol myo-inositol were measured at higher relative amounts in females. Note that the difference spectra in Fig. 2 span the range of 6.5–0.5 ppm, where resonances occur for the metabolites discussed here; full-range spectra (9.5–0.5 ppm) can be found in Supplementary Fig. S4.

To compare relative metabolite differences in the serum metabolomes across species, we constructed t-test filtered difference spectra by subtracting the average male Russian sturgeon spectrum from the average male Siberian sturgeon spectrum (Supplementary Fig. S5a); likewise for the females (Supplementary Fig. S5b). Species differences observed for both males and females (Supplementary Fig. S5) include higher levels of glucose and multiple amino acids in the Russian sturgeon metabolome. Note also that, for both males and females, we saw a pronounced overabundance in certain lipoproteins in Siberian sturgeon, although other lipoproteins, observed at slightly different ppm values, were somewhat more abundant in the Russian sturgeon metabolome.

### Epidermal mucous metabolome characterization for Russian and Siberian sturgeon

The low molecular weight endogenous metabolites identified by NMR in the epidermal mucus of Russian sturgeon and Siberian sturgeon are presented in Supplementary Tables S4 and S5, respectively. Like the blood serum metabolome, the mucous metabolome is also quite diverse, containing a variety of metabolite classes including amino acids, amines, nucleotides, organic acids, osmolytes, and carbohydrates. Particularly notable is a high relative concentration of xanthurenate (a quinoline carboxylic acid) in the epidermal mucus of Russian sturgeon (metabolite #59 in Supplementary Fig. S6), as well as high concentrations of myo-inositol in both species (metabolite #45 in Supplementary Figs. S6 and S7). (Note that the key that associates the spectrum peak-label numbers in Figs. S6 and S7 with the metabolites that give rise to the peaks is in Supplementary Table S3.)

**Fig. 3:**
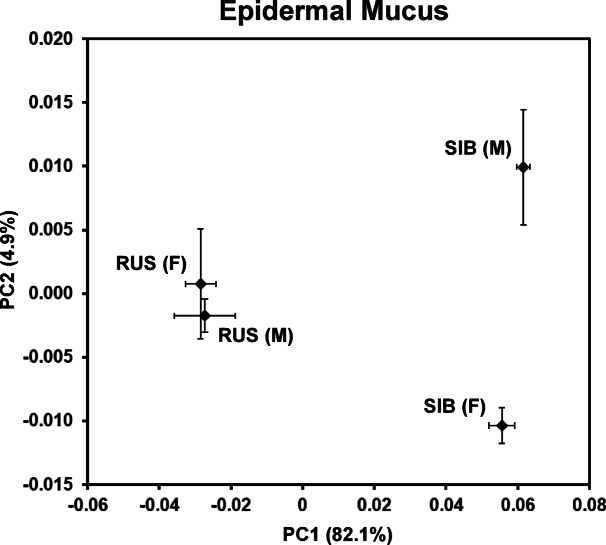
Score plot from principal components analysis (PCA) that compared epidermal mucus ^1^H NMR spectra from each sampled sturgeon species and sex. Markers are mean score values for the first two principal components (PC1 and PC2). Error bars represent standard errors of the means. The percent of variance in the dataset captured by each component is shown parenthetically in the axis titles. RUS, Russian sturgeon; SIB, Siberian sturgeon; M, male; F, females.

The epidermal mucous metabolome was evaluated in a similar fashion to that used for blood serum. A score plot from the PCA model that compared the mucous metabolomes from the male and female Russian and Siberian sturgeon is shown in Fig. 3. In agreement with the results of the blood serum analysis presented in Fig. 1, the greatest difference in mucous metabolomes is observed when comparing Russian sturgeon with Siberian sturgeon. Specifically, scores for both males and females are significantly different along PC1 when comparing across the species (*p* = 3.9E−06 and 2.1E−09, respectively). However, unlike the serum metabolome results, the mucous metabolomes of the male and female Siberian sturgeon differ to a greater extent (*p* = 0.0063 along PC2) than do the metabolomes of male and female Russian sturgeon (*p* = 0.6048 along PC2).

**Fig. 4:**
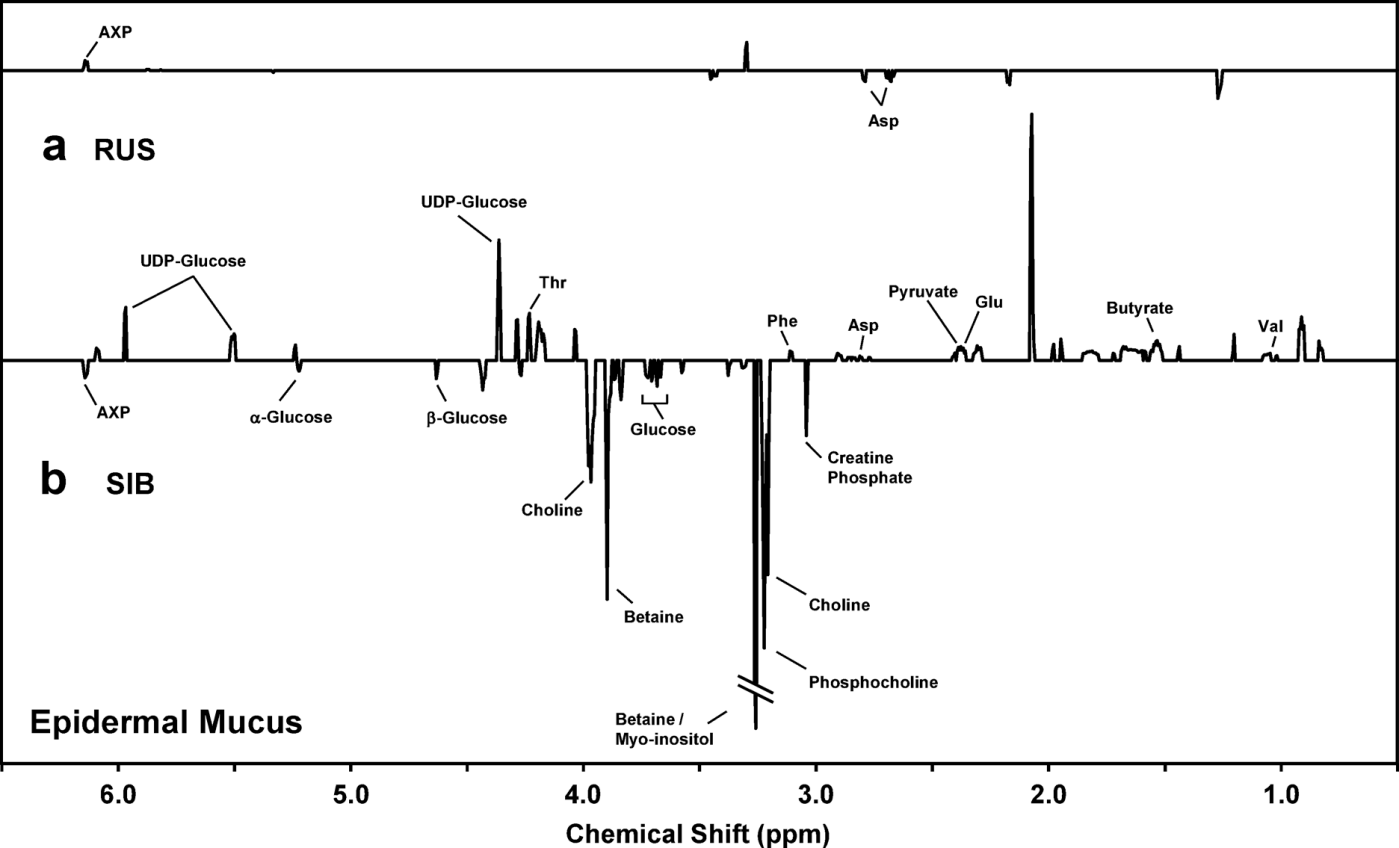
Average ^1^H NMR difference spectra, over the region 6.5–0.5 ppm, that compare the relative abundances of endogenous metabolites measured in the epidermal mucus of: (**a**) male versus female Russian sturgeon, and (**b**) male versus female Siberian sturgeon. Positive-going peaks represent metabolites that are relatively more abundant in females, while negative-going peaks represent those that are relatively more abundant in males. Only those peaks for which differences were determined to be significant (Student’s *t*-test, *p* < 0.05, see “Materials and methods” section for details) are included. Note that the two difference spectra are displayed using the same Y-axis scale, and that the negative-going peak for betaine/myo-inositol (3.26 ppm) in the bottom spectrum is off scale. These spectra are shown over the full range of 9.5–0.5 ppm in Supplementary Fig. S8. RUS, Russian sturgeon; SIB, Siberian sturgeon; Asp, aspartate; AXP, adenosine phosphate(s); Glu, glutamate; Phe, phenylalanine; Thr, threonine; UDP, uridine diphosphate; Val, valine.

Consistent with the PCA results, the t-test filtered difference spectrum that compares the male and female Russian sturgeon mucous metabolomes (Fig. 4a) reveals only a few relatively small peaks, reflecting comparatively minor metabolite differences. In contrast, the Siberian sturgeon difference spectrum is well populated, and several peaks exhibit considerable intensity (Fig. 4b). Most striking are higher relative abundances of betaine, choline, and phosphocholine in male Siberian sturgeon, as compared to the females (observed as negative-going peaks in Fig. 4b). Male Siberian sturgeon also displayed greater amounts of glucose and adenosine phosphate(s) (AXP) relative to females. Female Siberian sturgeon displayed higher relative amounts of uridine diphosphate glucose (UDP-glucose) and multiple amino acids. Note that the difference spectra in Fig. 4 span the range of 6.5–0.5 ppm, where resonances occur for the metabolites discussed here; full-range spectra (9.5–0.5 ppm) can be found in Supplementary Fig. S8.

Comparing the mucous metabolome across species (within each sex) using *t*-test filtered difference spectra (Supplementary Fig. S9) reaffirmed the large difference in the species-specific mucous metabolomes that was observed in the score plot in Fig. 3. Notably, sex had relatively little impact on inter-species differences as Supplementary Fig. S9a (male Siberian vs. male Russian) and Supplementary Fig. S9b (female Siberian vs. female Russian) appear highly similar. Evident from Supplementary Fig. S9 are large inter-species differences in xanthurenate and myo-insitol, with xanthurenate displaying higher relative abundances in Russian sturgeon (negative-going peaks), and myo-inositol more abundant in Siberian sturgeon (positive-going peaks).

### Extent of sturgeon metabolome sex specificity as compared to that of a sexually dimorphic fish species

To provide a wider context for some of these results, we compared the magnitude of sex specificity of the Russian and Siberian sturgeon blood serum and epidermal mucous metabolomes with those of the fathead minnow, which exhibits readily observable secondary sex characteristics and consistently displays sex specificity in metabolomes from a variety of sampled tissues and biofluids^4,11,19^. This comparison was accomplished, as described in the “Materials and methods” section, by determining the total intensity values for t-test filtered difference spectra using the summed absolute values of the magnitudes for all NMR spectral bins that were significantly different (*p* < 0.05) when comparing males to females. This total intensity value (reflecting the extent of sex specificity) was calculated for each available biological matrix for each of the three species.

**Fig. 5:**
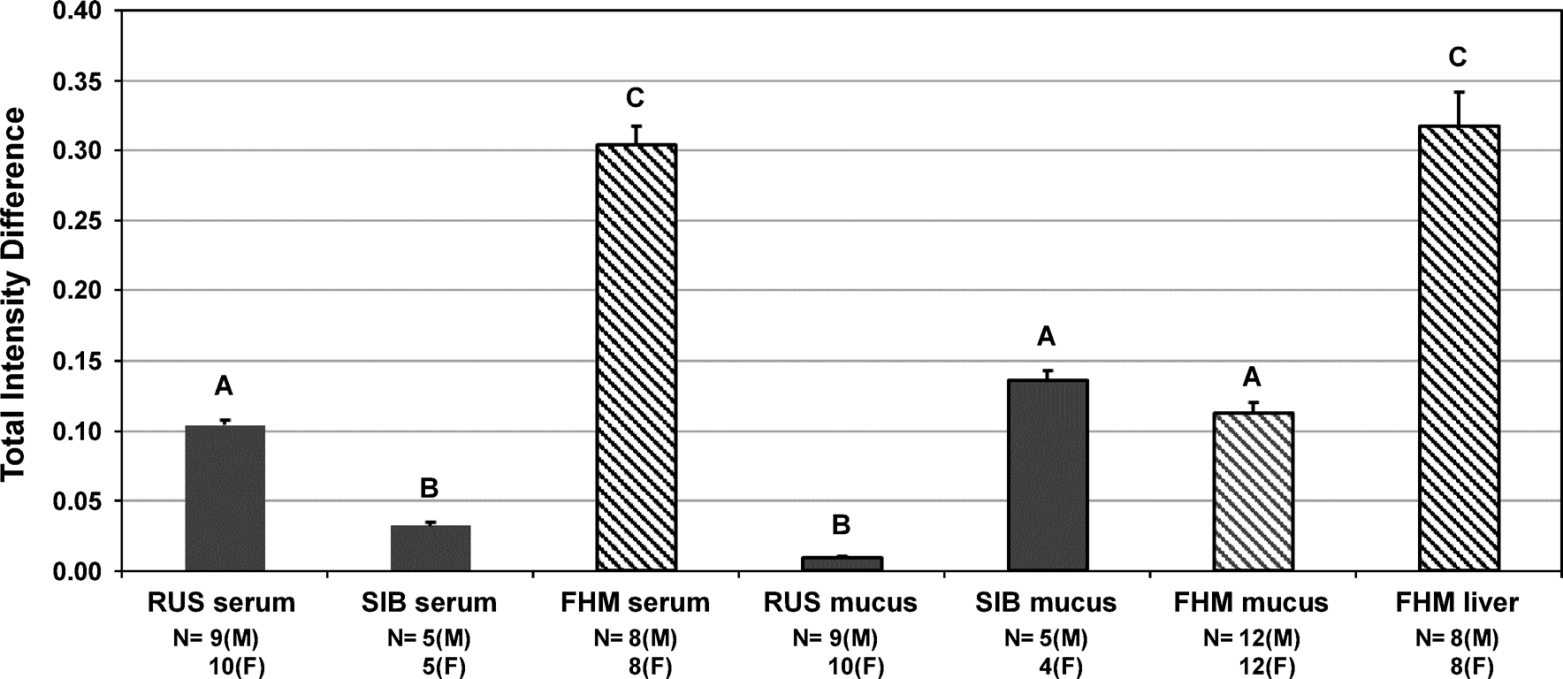
Total intensity difference values for estimating dissimilarity between male and female metabolomes (mean ± standard error) for various fish species and sample types, as measured using ^1^H NMR spectra (see “Materials and methods” section for details). Bars with different letters indicate significant differences based on ANOVA with posthoc Tukey’s test, *p* < 0.05. RUS, Russian sturgeon; SIB, Siberian sturgeon; FHM, fathead minnow. The number (N) of individuals, male (M) and female (F), included in the comparison is listed under each bar.

As shown in Fig. 5, the Russian sturgeon blood serum metabolome displayed greater sex specificity (i.e., a greater total intensity value) than that of the Siberian sturgeon, which is consistent with the serum PCA score plot (Fig. 1) and *t*-test filtered difference spectra (Fig. 2). However, the sex specificity displayed by the fathead minnow serum metabolome was considerably greater than that of either of the sturgeon species. On the other hand, while the sex specificity of the fathead minnow epidermal mucous metabolome was considerably greater than that of the Russian sturgeon, it was statistically indistinguishable (based on ANOVA with *p* < 0.05) from that of the Siberian sturgeon. Moreover, the magnitude of the sex-specificity of the Siberian sturgeon epidermal mucous metabolome was 43% of that measured for fathead minnow liver, a tissue known for its high level of sexual dimorphism, particularly in oviparous animals^20^.

## Discussion

Fisheries researchers and managers could benefit greatly from a technique for informing sturgeon health assessments, that relies on rapid and minimally invasive sample collection. As discussed earlier, NMR-based metabolomics using serum and epidermal mucus is an excellent candidate for such a technique. While metabolomic studies with fish have been well documented, many of these studies have primarily employed samples that required lethal means of collection and focused exclusively on determining the impacts from exposures to external stressors (e.g., heat stress, oxidative stress, exposure to chemical pollutants, etc.)^21–23^ In contrast, the long-term goal for this work is to use minimally invasive techniques to assess the health of wild populations of species, both generally and with regard to sex, to support restoration and reintroduction efforts. However, before field-deployment, an approach such as this is best developed using experimental designs with greater control than is achievable in the field. As a first step, we utilized a facility that housed a population of hatchery-raised adult males and females of two sturgeon species, whose ages and sexes had been established prior to our involvement. This facility offered a near-unique opportunity for collecting sturgeon serum and epidermal mucus under controlled experimental (e.g., environmental, dietary, etc.) conditions.

It is important to note that, while Russian and Siberian sturgeon are not (to our knowledge) of primary concern to any U.S. fisheries managers, they are, among all sturgeon species, closely related phylogenetically to several species that are. For example, when conducting phylogenetic analysis with complete mitochondrial genomes, Shen et al. recently found that the 21 extant sturgeon species they studied were distributed among four clades^24^. Russian and Siberian sturgeon were in the same clade as seven other species, including shortnose sturgeon (*Acipenser brevirostrum*) and lake sturgeon (*Acipenser fulvescen*s), both of which are of keen interest to US fisheries managers. Note that, NOAA (National Oceanic and Atmospheric Administration) Fisheries has an extensive program devoted to protection and recovery of shortnose sturgeon, which is found in rivers and bays along the eastern seaboard of North America and is listed as endangered throughout its range by the United States Endangered Species Act^25^. Furthermore, several US states are committed to conservation of lake sturgeon, which is found mainly in the Great Lakes and in Mississippi River drainages and is imperiled through much of its range. For example, since 2002, the Georgia Department of Natural Resources has been engaged in a long-term program to reintroduce lake sturgeon in the Coosa River basin, where it became locally extinct around 1960^3^.

In addition to providing information that may also be relevant to other sturgeon species of concern to US fisheries managers, it is noteworthy that the US Fish and Wildlife Service proposed listing Russian sturgeon, along with three other Eurasian sturgeons, as endangered under the Endangered Species Act^26^. Additionally, Russian and Siberian sturgeon are currently designated as critically endangered globally^2^. Thus, while these species were selected for this initial evaluation based on having been reared and maintained in a controlled setting and were largely viewed as potential surrogates for other sturgeon species, the results also support inclusion of metabolomic tools in the monitoring of endangered Russian and Siberian sturgeons in their native waters.

### The potential of blood serum-based metabolomics for sturgeon monitoring

The wide range of metabolites that we detected in Russian and Siberian sturgeon blood serum is generally consistent with a previous report on the NMR-detectable blood serum metabolome of Persian sturgeon (*Acipenser persicus*)^27^ as well as other fish species (and vertebrates in general), thus supporting the potential for routine use of the blood serum metabolome for monitoring numerous aspects of sturgeon health. Indeed, the blood metabolome has been used to expand understanding of salinity adaption in Persian sturgeon^27^ and reproductive development in Chinese sturgeon (*Acipenser sinensis*)^28,29^. Additionally, based on reports with other fish species (primarily salmonids), the blood metabolome may inform various other aspects of sturgeon physiology, including the immune response^30^ chemical toxicity^31^ fasting^32^ and temperature fluctuations^33^. Furthermore, circulating lipoproteins have previously been studied within the context of fish development and stress, including recently with sturgeon^34,35^ (although not using NMR spectroscopy). Given the relatively high lipid content of sturgeon blood relative to many other vertebrates^36^ and the varied roles lipids play in fish physiology (growth, reproductive development, spawning, etc.)^37^ lipids, apolipoproteins, and lipoproteins—to which the majority of blood lipids are bound—may present particularly informative endpoints for sturgeon monitoring.

In addition to lipoproteins, acute phase proteins (APPs) are also frequently detected in blood serum/plasma samples using NMR and can be used to monitor the status of the acute phase response^38,39^ a primordial mechanism for innate immunity that responds to a variety of biological perturbations (e.g., infection, injury, neoplastic growth)^40^. APPs represent many structurally and functionally diverse proteins and have been widely utilized in human health monitoring. For example, a variety of APPs have been closely associated with human inflammation^41^ wherein the concentrations of so-called “positive APPs” increase with inflammation while the concentrations of “negative APPs” decrease^41^. Recent studies have also revealed the significance of APPs for sturgeon monitoring; indeed, changes in levels of serum amyloid A (an evolutionarily conserved APP) have been reported in sturgeon in response to both bacterial challenge (50-fold increase) and thermal stress (3-fold decrease)^35,42^.

NMR spectra of human plasma and serum contain a prominent and well-documented resonance (i.e., peak) that arises from numerous glycan residues on a variety of enzymatically glycosylated APPs^39^. This spectral feature, termed GlycA (occurring near 2.0 ppm), has been found to increase in intensity during acute and chronic inflammation and has been successfully promoted as a composite biomarker for risk or progression of a number of human diseases including rheumatoid arthritis^43^ and cardiovascular disease^44^. Given its ubiquity in human serum NMR spectra, and the recognized evolutionary conservation of the APR; as anticipated, we observed this feature in the sturgeon serum spectra in the current study (see metabolite #24 in Supplementary Figs. S1 and S2). It is important to note that, in humans and likely in other species, a variety of major APPs contribute to the composite GlycA NMR signal, and that changes in this signal (e.g., as a function of the inflammation response) are due not only to changes in individual APP concentrations, but also to their specific glycan structures, which are modified, for example, by circulating glycosidases^39^. Thus, given this complexity, we were not readily able here to assign the specific APPs that contributed to the GlycA feature that we observed. However, recent developments, including those employing diffusion-edited NMR along with spectral deconvolution, have shown promise for precisely quantifying specific APPs in human serum^38^. In view of the important role of APPs in the vertebrate stress response, their tentative identification in the serum of sturgeon species should help encourage further efforts to monitor captive and wild stocks of sturgeon, using minimally invasive blood collection. More broadly, these findings highlight the utility of NMR spectroscopy for simultaneous and rapid measurement of a range of components (e.g., amino acids, carbohydrates, proteins, lipids, etc.) in sturgeon blood that have relevance in vertebrate physiology.

Given their extensive physiological relevance, it is perhaps not surprising that many of these measurements displayed sex specificity as evident from the PCA score plot evaluating sex (and species) differences in the blood serum NMR spectra (Fig. 1). This sex-specificity is particularly striking for Russian sturgeon, with males and females clearly separating along principal component two, despite a larger portion of the variation being attributable to species differences along principal component one. While the relative abundance of lactate in female Russian sturgeon (as compared to the males) appears to contribute heavily to this species’ sex-specificity, several additional metabolites and proteins contribute as well (Fig. 2a). This contrasts with Siberian sturgeon, for which the separation in the mean score values for males and females did not meet the threshold for statistical significance (*p* < 0.05) along either of the two principal components in the PCA score plot (Fig. 1). However, using t-test filtered difference spectra, we were able to identify several individual metabolites that show a statistically significant difference in relation to sex for Siberian sturgeon (Fig. 2b). Furthermore, the sex-specificity of many of these metabolites were also observed with Russian sturgeon (Fig. 2a). For example, resonances from glucose and certain lipoproteins display greater relative abundance in male sturgeon of both species (relative to the females), while female sturgeon of both species display greater relative abundances of myo-inositol and glutamine (Fig. 2a and b).

Finally, there has been increasing interest recently in using metabolomics with fish for cross-species health assessments with a focus on identifying metabolite-based markers that indicate stress and disease, or which are generally predictive of deviations from homeostasis^10,45,46^. The substantial differences in the blood serum metabolomes between the two sturgeon species studied here, as illustrated in the score plot of Fig. 1, suggests that there may be distinct differences in this metabolome across other (if not all) sturgeon species. As a result, it may be possible to identify and utilize specific metabolite and protein markers that are particularly diagnostic for a specific species. Conversely, such specificity might also suggest that it will be challenging to discover metabolite/protein markers that can be broadly applied for health monitoring of multiple sturgeon species. However, it is important to note that the score plot in Fig. 1 is based on the whole metabolome. While this composite measurement of all NMR-detectable endogenous metabolites may exhibit significant differences across all sturgeon species, it is possible if not likely—given the high information content of the metabolome—that certain individual metabolites will respond similarly and systematically to a given stress or disease across multiple species.

### The potential of epidermal mucous-based metabolomics for sturgeon monitoring

We and others have found the mucous metabolome to be remarkably complex regarding the number and classes of detectable metabolites across a variety of fish species^46^ presumably a result of the many and varied roles that epidermal mucus plays in fish biology^47^. The Russian and Siberian sturgeon epidermal mucous metabolomes appear to be no exception. Also consistent with these earlier reports is the presence of some notable metabolites/metabolite classes in sturgeon epidermal mucus that may offer particularly useful markers of stress/disease. This includes several organic osmolytes (betaine, myo-inositol, taurine, and trimethylamine-N-oxide) that were observed in the metabolomes of both species, suggesting the utility of epidermal mucus for monitoring osmotic stress in sturgeons. This is encouraging with respect to monitoring wild sturgeons for physiological changes that result from exposure to environmental stressors. For example, climate change is anticipated to alter the hydrologic properties of estuaries and rivers (that multiple sturgeon species depend upon), by producing rapid changes in salinity due, in part, to altered timing of seasonal snowmelt runoff and increased drought severity^48,49^. Measuring osmolytes in epidermal mucus may provide regulators and fisheries managers with a rapid and non-lethal tool for monitoring at-risk sturgeon species to inform restoration/protection efforts in affected waters. Additionally, mucous metabolites that reflect energetic status may prove valuable for conducting general (or non-specific) assessments of sturgeon population heath. Indeed, several metabolites related to cellular energetics were measured in these sturgeon species, including adenosine phosphate(s) (AXP), amino acids, citrate, creatine, creatine phosphate (Russian sturgeon only), glucose (Siberian sturgeon only), guanosine/uridine triphosphate/monophosphate (GXP/UXP), lactate, pyruvate, and uridine diphosphate-glucose (UDP-glucose) (Siberian sturgeon only). Finally, glutamate and glutamine are of enormous physiological importance in fish, controlling functions as diverse as ammonia balance, metabolism, antioxidant capacity, and neurotransmission^50^. These amino acids were readily measured in the epidermal mucus of both species and may offer additional markers for detecting a range of adverse effects resulting from exposures to impacted surface waters.

Blood plasma/serum collection, despite also being non-lethal, requires a greater amount of time and handling (i.e., is more invasive) than mucus. Therefore, it has been suggested that the extensive biochemical information contained in the mucous metabolome may offer opportunities for its use as a strong complement, or, perhaps even in some cases, a substitute or surrogate for blood monitoring with fish^4,51^. With this in mind, we compared the blood serum and epidermal mucous metabolomes to determine the extent of overlap with respect to identified metabolites and found that, for both species, a considerably greater proportion of these metabolites were shared between serum and mucus than were unique to one or the other biofluid (Supplementary Tables S6 and S7). While there are limits to what can be concluded from these analyses, the physiological relevance of the high degree of overlap in endogenous metabolites suggest the prospect of some level of useful surrogacy. For example, note that Hajirezaee et al. reported decreases in the levels of blood osmolytes (e.g., taurine and betaine) in Persian sturgeon in response to salinity challenge^52^. Recall, that we detected these and other osmolytes in both the serum and mucus metabolomes of Russian and Siberian sturgeons. Clearly, investigating osmolytes in epidermal mucous in comparison to blood osmolytes when monitoring for osmotic stress in sturgeons is warranted.

It was previously determined that some fish species (e.g., fathead minnow and rainbow smelt (*Osmerus mordax*)), displayed sex-specificity in the composition of their epidermal mucous metabolome, providing potentially critical information on sex-dependent characteristics^4,46^. The observation in the current study that the sturgeon mucous metabolome also displayed sex-specificity is particularly encouraging given the low degree of sexual dimorphism in sturgeons. Interestingly, the magnitude of this specificity appeared here to be species dependent—the mucus of Siberian sturgeon displayed greater sexual dimorphism than did the Russian sturgeon (Fig. 3). Whether this phenomenon is inherent to these species or whether differences in some other factor(s) are exerting influence (e.g., maturity, reproductive status, etc.), will require further investigation. Interestingly, note in the “Materials and methods” section that males and females were from the same year cohort in the case of the sampled Russian sturgeons (2011), while for Siberian sturgeon, the sampled females were 2006-cohort fish, and the males were 2003-cohort fish. However, it seems unlikely that this relatively small difference in age is a dominant contributor to the observed differences in the mucous metabolome of male and female Siberian sturgeon. Indeed, once sturgeon reach maturity (as all these sampled fish had), they engage in the same behaviors for decades; thus, a relatively small difference in age among mature fish would likely not result in large differences in their metabolomes. Furthermore, if this difference in age between male and female Siberian sturgeon was contributing to the sex specificity seen with mucus (Fig. 3), then this was notably not the case with serum; indeed, the sex specificity with serum was considerably greater for Russian sturgeon (where males and females were the same age) than for Siberian sturgeon (Fig. 1). Note that we are currently conducting studies that incorporate juvenile sturgeons to assess the age dependency of this approach, an important consideration for assessing wild populations.

Finally, it is also worth noting that the *species* specificity of the epidermal mucous metabolome was greater than that measured for the blood serum for these species. This is suggested by comparing the score plot from the PCA model for the mucus versus that for serum. Specifically, note the greater separation between species along the first principal component for mucus (Fig. 3) as compared to serum (Fig. 1), as well as a higher percent variation in the data captured by the first principal component (82.1% for mucus vs. 50.0% for serum). This greater level of species specificity for mucus, suggested by the PCA score plots, was further investigated by calculating the total intensity values for the t-test filtered difference spectra that compared the metabolomes of the species for mucus (Supplementary Figure S9) and serum (Supplementary Figure S5). A bar graph of these total intensity values (Supplementary Figure S10; generated in the same manner as that of Fig. 5) confirms that the species specificity is significantly greater in the case of the mucous metabolome as compared to that of serum. Thus, in agreement with previous reports^46^ the epidermal mucus may prove to be species-specific in sturgeons as well and potentially able to capture species-specific traits or responses that go undetected in blood serum/plasma.

### Further considerations for developing non-lethal metabolomic tools for monitoring wild sturgeons

Given the current and potentially increasing restrictions on the handling and sample collection from many sturgeon species, the rich biochemical and physiological information that can be provided non-lethally by metabolomics—using serum and epidermal mucus—offers a timely and valuable toolset for expanding monitoring efforts. Additionally, the possibility of sex-specific health monitoring in sturgeons with minimally invasive methods provides an opportunity to increase the effectiveness of wild population monitoring efforts. It is particularly encouraging that we found sex-based biases in the abundances of some metabolites that were consistent across the studied species. This suggests that these observed sex differences are potentially species-independent and might be expected to be conserved in wild fish, thus, potentially providing a generally applicable and information-rich tool for sex-specific fish health monitoring. Additionally, the documented responsivity of the blood serum/plasma and epidermal mucous metabolomes to various environmental stressors in other fish species^4,53^ suggests that samples from sturgeon populations that reside in polluted waters (e.g., spawning adults in urban river systems) may offer the possibility of an “early warning” of adverse effects, thereby providing time for preventative steps before reproduction and/or survival are impacted. To enable these efforts, further investigations are needed to improve understanding of the health implications associated with varying levels of blood metabolites/proteins (e.g., acute phase proteins) and epidermal mucous metabolites (e.g., osmolytes) in sturgeons, as well as sources of variability (e.g., temperature, water quality, diet, etc.) that might confound interpretation. Additionally, this approach could be expanded to the assessment of various other vulnerable fish species that are feasible to sample in this fashion, including those identified as most critically endangered. Ideally, blood and mucus collection could be widely included in ongoing fish monitoring efforts to best leverage resources and minimize further impacts on sensitive populations. In our experience, the added burden of collecting epidermal mucus as part of ongoing state and federal monitoring efforts is negligible. Blood collection is relatively more involved and will benefit from the incorporation of methods more suitable for the field. Regardless, while careful consideration is required to prioritize next steps, the great potential for non-lethal metabolomics-based monitoring of sturgeons presented here suggests that investments in time and resources to pursue these additional lines of investigation would be richly rewarded.

## Supplementary Information

Below is the link to the electronic supplementary material.

Supplementary Material 1

## Data Availability

Data reported are available in the U.S. Environmental Protection Agency’s Science-Hub database (http://catalog.data.gov/organization/epa-gov).
