# Supplementary material for: Evaluation of minimally invasive metabolomic methods for assessing the health of sturgeons
Source: Sci Rep. 2025 Oct 2;15:34461. doi: 10.1038/s41598-025-17654-2 (PMC12491608; doi:10.1038/s41598-025-17654-2)
Supplement: Supplementary file 1 — Supplementary Material 1 [file 41598_2025_17654_MOESM1_ESM.pdf]

Evaluation of Minimally Invasive Metabolomic Methods for Assessing the Health of Sturgeons

Timothy W. Collette[a], Shannon N. Romano[a], Quincy Teng[a], Adam G. Fox[b], J. Shane Kornberg[b], and Drew R. Ekman[a]*

[a] U.S. EPA, Center for Environmental Measurements and Modeling, Athens, GA, USA

[b] Warnell School of Forestry and Natural Resources, University of Georgia, Athens, GA, USA

*To whom correspondence should be addressed at Ecosystem Processes Division, U.S. Environmental Protection Agency, 960 College Station Rd., Athens, GA 30605

Email: ekman.drew@epa.gov

SUPPLEMENTARY TABLES –

Supplementary Table S1: Low molecular weight endogenous metabolites and lipoprotein lipid resonances identified by NMR for blood serum from male and female Russian sturgeon. Chemical formulas in bold indicate the functional group responsible for the observed peak.

**Endogenous Metabolites**	**Chemical Shift (ppm)**
Acetate	1.92
Acetone	2.23
Acetoacetate	2.28, 3.43
GlycA	2.03
Alanine (Ala)	1.48, 3.78
Arginine (Arg)	1.65, 1.71, 1.91, 3.23, 3.78
Aspartate (Asp)	2.64, 2.76, 3.87
Betaine	3.27, 3.90
Butyrate	0.89, 1.55, 2.14
Cholesterol (CHO)	See “Lipids” below
Citrate	2.53, 2.68
Creatine	3.06, 3.94
Creatine phosphate	3.09, 3.95
Cysteine (Cys)	3.03, 3.07, 3.94
Formate	8.46
Glucose	3.25, 3.41, 3.47, 3.49, 3.54, 3.73, 3.77, 3.84, 3.90, 4.65, 5.24
Glutamate (Glu)	2.05, 2.12, 2.34, 3.75
Glutamine (Gln)	2.14, 2.46, 3.77
Glycerol	3.55, 3.65, 3.78
Glycine (Gly)	3.56
Histidine (His)	3.14, 3.27, 4.00, 7.08, 7.84
Isoleucine (Ile)	0.93, 1.01, 1.25, 1.47, 1.98, 3.67
Lactate	1.33, 4.12
Leucine (Leu)	0.96, 1.69, 1.75, 3.74
Lysine (Lys)	1.73, 1.91, 3.02, 3.75
Methionine (Met)	2.14, 2.20, 2.64, 3.85
Myo-Inositol	3.26, 3.54, 3.63, 4.06
Phenylalanine (Phe)	3.13, 3.29, 4.00, 7.33, 7.38, 7.43
Phosphocholine	3.22
Proline	2.02, 2.06, 2.35, 3.34, 3.40, 4.11
Pyruvate	2.38
Serine (Ser)	3.84, 3.94, 3.99
SPC ($-^{+}N(CH_3)_3$)	3.23
Taurine	3.26, 3.42

Threonine (Thr)	1.33, 3.59, 4.25
Tryptophan (Trp)	3.30, 3.46, 4.07,7.19, 7.28, 7.54, 7.73
Tyrosine (Tyr)	3.03, 3.19, 3.94, 6.90, 7.19
Valine (Val)	0.99, 1.04, 2.28, 3.61
Lipoprotein Lipids (L.L.)	0.66-0.72 (Cholesterol (CHO) C18 $\mathbf{CH_3}$)
	0.82-0.91 ($-CH_2-\mathbf{CH_3}$)
	0.92-0.96 (Cholesterol (CHO) $\mathbf{CH_2}$)
	0.98-1.04 (Cholesterol (CHO) C19 $\mathbf{CH_3}$)
	1.28-1.31 ($-CH_2-\mathbf{CH_2}-CH_2-$)
	1.50-1.62 ($-\mathbf{CH_2}-CH_2-CO$)
	1.95-2.02 ($-\mathbf{CH_2}-CH=CH-$)
	2.18-2.28 ($-\mathbf{CH_2}-CO$)
	2.76-2.83 ($-CH=CH-\mathbf{CH_2}-CH=CH-$)
	4.20-4.30 ($-C\mathbf{H_2}-O-$)
	5.26-5.36 ($-\mathbf{CH}=\mathbf{CH}-$)

Key: GlycA, glycosylated acute phase protein A; SPC, supramolecular phospholipid composite.

Supplementary Table S2: Low molecular weight endogenous metabolites and lipoprotein lipid resonances identified by NMR for blood serum from male and female Siberian sturgeon. Chemical formulas in bold indicate the functional group responsible for the observed peak.

**Endogenous Metabolites**	**Chemical Shift (ppm)**
Acetone	2.23
Acetoacetate	2.28, 3.43
GlycA	2.03
Alanine (Ala)	1.48, 3.78
Arginine (Arg)	1.65, 1.71, 1.91, 3.23, 3.75
Aspartate (Asp)	2.64, 2.76, 3.87
Betaine	3.27, 3.90
Butyrate	0.89, 1.55, 2.14
Cholesterol (CHO)	See “Lipids” below
Citrate	2.53, 2.68
Creatine	3.06, 3.94
Creatine phosphate	3.09, 3.95
Cysteine (Cys)	3.03, 3.07, 3.94
Formate	8.46
Glucose	3.25, 3.41, 3.47, 3.49, 3.54, 3.73, 3.77, 3.84, 3.90, 4.65, 5.24
Glutamate (Glu)	2.05, 2.12, 2.34, 3.75
Glutamine (Gln)	2.14, 2.46, 3.76
Glycerol	3.56, 3.65, 3.78
Glycine (Gly)	3.56
Histidine (His)	3.14, 3.27, 4.00, 7.08, 7.84
Isoleucine (Ile)	0.93, 1.01, 1.25, 1.47, 1.98, 3.67
Lactate	1.33, 4.12
Leucine (Leu)	0.96, 1.69, 1.75, 3.74
Lysine (Lys)	1.73, 1.89, 3.02, 3.75
Methionine (Met)	2.14, 2.20, 2.64, 3.85
Myo-Inositol	3.26, 3.54, 3.63, 4.06
Phenylalanine (Phe)	3.13, 3.29, 4.00, 7.33, 7.38, 7.43
Phosphocholine	3.22
Proline	2.01, 2.07, 2.35, 3.34, 3.40, 4.11
Pyruvate	2.38
Serine (Ser)	3.85, 3.95, 3.99
SPC ($-^{+}N(CH_3)_3$)	3.23
Taurine	3.26, 3.42
Threonine (Thr)	1.33, 3.59, 4.25
Tryptophan (Trp)	3.30, 3.46, 4.07,7.19, 7.28, 7.54, 7.73
Tyrosine (Tyr)	3.03, 3.19, 3.94, 6.90, 7.19
Valine (Val)	0.99, 1.04, 2.28, 3.61

Lipoprotein Lipids (L.L)	0.66-0.72 (Cholesterol (CHO) C18 **CH_3**)
	0.82-0.91 (-CH_2-**CH_3**)
	0.92-0.96 (Cholesterol (CHO) **CH_2**)
	0.98-1.04 (Cholesterol (CHO) C19 **CH_3**)
	1.28-1.31 (-CH_2-**CH_2**-CH_2-)
	1.50-1.62 (-**CH_2**-CH_2-CO)
	1.95-2.02 (-**CH_2**-CH=CH-)
	2.18-2.28 (-**CH_2**-CO)
	2.76-2.83 (-CH=CH-**CH_2**-CH=CH-)
	4.20-4.30 -C**H_2**-O-)
	5.26-5.36 (-**CH**=**CH**-)

Key: GlycA, glycosylated acute phase protein A; SPC, supramolecular phospholipid composite.

Supplementary Table S3: Key that associates low molecular weight endogenous metabolites (and other chemicals) with the labels of the NMR peaks that they give rise to in the spectra that appear in Supplementary Figures S1, S2, S6, and S7. Chemical formulas in bold indicate the functional group responsible for the observed peak.

Peak-label Number	Associated Metabolite or Other Chemical
1	Acetate
2	Acetoacetate
3	Acetone
4	Alanine (Ala)
5	Arginine (Arg)
6	Asparagine (Asn)
7	Aspartate (Asp)
8	AXP
9	Betaine
10	Butyrate
11	Buffer Contaminant
12	Cholesterol (CHO) (C18 $\mathbf{CH_3}$)
13	Cholesterol (CHO) (C19 $\mathbf{CH_3}$)
14	Cholesterol (CHO) ($\mathbf{CH_2}$)
15	Choline
16	Citrate
17	Creatine
18	Creatine phosphate
19	Cysteine (Cys)
20	Formate
21	Glucose
22	Glutamate (Glu)
23	Glutamine (Gln)
24	GlycA (-NCO$\mathbf{CH_3}$)
25	Glycerol
26	Glycine (Gly)
27	GXP/UXP
28	Histamine
29	Histidine (His)
30	Isoleucine (Ile)
31	Isopropanol
32	Lactate
33	Leucine (Leu)
34	Lipoprotein Lipids (-**CH**=**CH**-)
35	Lipoprotein Lipids (-CH=CH-$\mathbf{CH_2}$-CH=CH-)
36	Lipoprotein Lipids (-$\mathbf{CH_2}$-CH=CH-)
37	Lipoprotein Lipids (-CH_2-$\mathbf{CH_2}$-CH_2-)
38	Lipoprotein Lipids (-$\mathbf{CH_2}$-CH_2-CO)

39	Lipoprotein Lipids (-CH_2-**CH_3**)
40	Lipoprotein Lipids (**-CH_2**-CO)
41	Lipoprotein Lipids (**-CH_2**-O)
42	Lysine (Lys)
43	Residual Methanol
44	Methionine (Met)
45	Myo-Inositol
46	o-phosphoethanolamine
47	Phenylalanine (Phe)
48	Proline
49	Pyruvate
50	Serine (Ser)
51	SPC (-+$N(CH_3)_3$)
52	Taurine
53	Threonine (Thr)
54	Trimethylamine N-oxide (TMAO)
55	Tryptophan (Trp)
56	Tyrosine (Tyr)
57	UDP-glucose
58	Valine (Val)
59	Xanthurenate
60	a-Glucose
61	b-Glucose
62	Residual H_20
63	Phosphocholine

Key: GlycA, glycosylated acute phase protein A; SPC, supramolecular phospholipid composite; AXP, adenosine phosphate(s); GXP/UXP, guanosine / uridine phosphate(s); UDP, uridine diphosphate.

Supplementary Table S4: Low molecular weight endogenous metabolites identified by NMR for epidermal mucus from male and female Russian sturgeon.

**Endogenous Metabolites**	**Chemical Shift (ppm)**
Acetate	1.92
AXP	6.15, 8.23, 8.58
Arginine (Arg)	1.65, 1.71, 1.91, 3.23, 3.75
Asparagine (Asn)	2.94, 4.00
Aspartate (Asp)	2.67, 2.81, 3.89
Betaine	3.26, 3.90
Butyrate	0.89, 1.54, 2.15
Choline	3.21, 4.03
Citrate	2.53, 2.67
Creatine	3.03, 3.93
Creatine phosphate	3.05, 3.93
Cysteine (Cys)	3.03, 3.06, 3.94
Formate	8.46
Glutamate (Glu)	2.05, 2.12, 2.34, 3.75
Glutamine (Gln)	2.14, 2.46, 3.76
Glycerol	3.57, 3.66, 3.79
Glycine (Gly)	3.56
GXP/UXP	5.97, 8.12
Isopropanol	1.17, 4.01
Lactate	1.33, 4.11
Myo-Inositol	3.26, 3.54, 3.62, 4.06
o-phosphoethanolamine	3.21, 3.97
Phosphocholine	3.22
Pyruvate	2.37
Serine (Ser)	3.83, 3.94, 3.99
Taurine	3.26, 3.42
Threonine (Thr)	1.33, 3.59, 4.25
Trimethylamine N-oxide (TMAO)	3.26
Tyrosine (Tyr)	3.03, 3.19, 3.94, 6.90, 7.19
UDP-glucose	3.78, 3.86, 4.14, 4.22, 4.29, 4.36, 5.51, 5.96, 5.98, 7.95
Xanthurenate	6.89, 7.13, 7.34, 7.50

Key: AXP, adenosine phosphate(s); GXP/UXP, guanosine / uridine phosphate(s); UDP, uridine diphosphate.

Supplementary Table S5: Low molecular weight endogenous metabolites identified by NMR for epidermal mucus from male and female Siberian sturgeon.

**Endogenous Metabolites**	**Chemical Shift (ppm)**
Alanine (Ala)	1.48, 3.78
AXP	6.15, 8.23, 8.58
Arginine (Arg)	1.65, 1.71, 1.91, 3.23, 3.74
Aspartate (Asp)	2.67, 2.81, 3.89
Betaine	3.26, 3.90
Butyrate	0.89, 1.54, 2.15
Choline	3.21, 3.98
Citrate	2.53, 2.69
Creatine	3.03, 3.91
Formate	8.46
Glucose	3.25, 3.41, 3.47, 3.49, 3.57, 3.72, 3.77, 4.65, 5.24
Glutamate (Glu)	2.05, 2.12, 2.34, 3.75
Glutamine (Gln)	2.14, 2.44, 3.75
Glycine (Gly)	3.56
GXP/UXP	5.97, 8.10
Histamine	3.02, 3.28, 7.08, 7.85
Isopropanol	1.17, 4.01
Lactate	1.33, 4.11
Methionine (Met)	2.14, 2.64, 3.85
Myo-Inositol	3.26, 3.54, 3.62, 4.06
o-phosphoethanolamine	3.21, 3.97
Phenylalanine (Phe)	3.11, 3.33, 4.98, 7.30, 7.40, 7.58
Phosphocholine	3.22
Pyruvate	2.37
Serine (Ser)	3.81, 3.92, 3.98
Taurine	3.26, 3.42
Threonine (Thr)	1.32, 3.54, 4.22
Trimethylamine N-oxide (TMAO)	3.26
Tyrosine (Tyr)	3.03, 3.19, 3.94, 6.90, 7.17
UDP-glucose	3.78, 3.86, 4.14, 4.22, 4.29, 4.36, 5.51, 5.96, 5.98, 7.95
Valine (Val)	0.98, 1.04, 2.27, 3.60
Xanthurenate	6.87, 7.11, 7.33, 7.49

Key: AXP, adenosine phosphate(s); GXP/UXP, guanosine / uridine phosphate(s); UDP, uridine diphosphate.

Supplementary Table S6: Association of low molecular weight endogenous metabolites with the biofluids from male and female Russian sturgeon in which they were detected with NMR spectroscopy.

**Biological Fluid**	**Endogenous Metabolite**
Serum and Epidermal Mucus	Acetate
	Arginine (Arg)
	Aspartate (Asp)
	Betaine
	Butyrate
	Citrate
	Creatine
	Creatine phosphate
	Cysteine (Cys)
	Formate
	Glutamate (Glu)
	Glutamine (Gln)
	Glycerol
	Glycine (Gly)
	Lactate
	Myo-Inositol
	Phosphocholine
	Pyruvate
	Serine (Ser)
	Taurine
	Threonine (Thr)
	Tyrosine (Tyr)
Serum (only)	Acetoacetate
	Acetone
	Alanine (Ala)
	Cholesterol (CHO)
	Glucose
	GlycA
	Histidine (His)
	Isoleucine (Ile)
	Leucine (Leu)
	Lipids
	Lysine (Lys)
	Methionine (Met)
	Phenylalanine (Phe)
	Proline
	SPC (-+N$(CH_3)_3$)
	Tryptophan (Trp)
	Valine (Val)

Epidermal Mucus (only)	Asparagine (Asn)
	AXP
	Choline
	GXP/UXP
	Isopropanol
	o-phosphoethanolamine
	Trimethylamine N-oxide (TMAO)
	UDP-glucose
	Xanthurenate

Key: GlycA, glycosylated acute phase protein A; SPC, supramolecular phospholipid composite; AXP, adenosine phosphate(s); GXP/UXP, guanosine / uridine phosphate(s); UDP, uridine diphosphate.

Supplementary Table S7: Association of low molecular weight endogenous metabolites with the biofluids from male and female Siberian sturgeon in which they were detected with NMR spectroscopy.

**Biological Fluid**	**Endogenous Metabolite**
Serum and Epidermal Mucus	Alanine (Ala)
	Arginine (Arg)
	Aspartate (Asp)
	Betaine
	Butyrate
	Citrate
	Creatine
	Formate
	Glucose
	Glutamate (Glu)
	Glutamine (Gln)
	Glycine (Gly)
	Lactate
	Methionine (Met)
	Myo-Inositol
	Phenylalanine (Phe)
	Phosphocholine
	Pyruvate
	Serine (Ser)
	Taurine
	Threonine (Thr)
	Tyrosine (Tyr)
	Valine (Val)
Serum (only)	Acetoacetate
	Acetone
	Cholesterol (CHO)
	Creatine phosphate
	Cysteine (Cys)
	GlycA
	Glycerol
	Histidine (His)
	Isoleucine (Ile)
	Leucine (Leu)
	Lipids
	Lysine (Lys)
	Proline
	SPC (-+N(CH_3)$_3$)
	Tryptophan (Trp)

Epidermal Mucus (only)	AXP
	Choline
	GXP/UXP
	Histamine
	Isopropanol
	o-phosphoethanolamine
	Trimethylamine N-oxide (TMAO)
	UDP-glucose
	Xanthurenate

Key: GlycA, glycosylated acute phase protein A; SPC, supramolecular phospholipid composite; AXP, adenosine phosphate(s); GXP/UXP, guanosine / uridine phosphate(s); UDP, uridine diphosphate.

SUPPLEMENTARY FIGURE LEGENDS –

Supplementary Figure S1. The ^{1}H NMR spectrum (prior to the normalization and residual water / solvent peak removal steps that are described in the Material and Methods section of the main manuscript) of blood serum from a representative (a) male and (b) female Russian sturgeon. The key that associates the spectrum-peak labels (i.e., the peak numbers) with the metabolites and proteins that give rise to the peaks is in Supplementary Table S3.

Supplementary Figure S2. The ^{1}H NMR spectrum (prior to the normalization and residual water / solvent peak removal steps that are described in the Material and Methods section of the main manuscript) of blood serum from a representative (a) male and (b) female Siberian sturgeon. The key that associates the spectrum-peak labels (i.e., the peak numbers) with the metabolites and proteins that give rise to the peaks is in Supplementary Table S3.

Supplementary Figure S3. Diffusion-edited ^{1}H NMR spectrum (prior to the normalization and residual water / solvent peak removal steps that are described in the Material and Methods section of the main manuscript) of blood serum from a representative male Russian sturgeon. NMR signals/peaks from molecules that diffuse quickly (e.g., low molecular weight metabolites) are minimized, while signals/peaks from molecules that diffuse slowly (e.g., higher molecular weight lipoproteins) are maximized. Flexible groups (e.g., lipids) attached to molecules that diffuse slowly are readily detected. The type of hydrogen (e.g., methyl) responsible for producing each peak is indicated parenthetically using underline (e.g., $C\underline{H}_3$). Key: C-19, carbon 19; C-18, carbon 18; GlycA, glycosylated acute phase protein A; L.L., lipoprotein lipid; SPC, supramolecular phospholipid composite.

Supplementary Figure S4. Average ^{1}H NMR difference spectra that compare the relative abundances of endogenous metabolites measured in the serum of: (a) male versus female Russian sturgeon, and (b) male versus female Siberian sturgeon. Positive-going peaks represent metabolites that are relatively more abundant in females, while negative-going peaks represent those that are relatively more abundant in males. Only those peaks for which differences were determined to be significant (Student's t-test, $p < 0.05$, see Materials and Methods section in the main manuscript for details) were included. Note that the two difference spectra are displayed using the same Y-axis scale, and that the positive-going peak for lactate (1.33 ppm) in the top spectrum is off scale. The type of hydrogen (e.g., methyl) responsible for producing each peak is indicated parenthetically using underline (e.g., $C\underline{H}_3$). Key: RUS, Russian sturgeon; SIB, Siberian sturgeon; Ala, alanine; Arg, arginine; Asp, aspartate; Cys, cysteine; GlycA, glycosylated acute phase protein A; Gln, glutamine; Ile, isoleucine; Leu, leucine; L.L., lipoprotein lipids; Lys, lysine; Met, methionine; Phe, phenylalanine; Thr, threonine; Tyr, tyrosine.

Supplementary Figure S5. Average ^{1}H NMR difference spectra that compare the relative abundances of endogenous metabolites measured in the serum of Russian sturgeon versus Siberian sturgeon for (a) males of both species and (b) females of both species. Positive-going peaks represent metabolites that are relatively more abundant for Siberian sturgeon, while negative-going peaks represent those that are relatively more abundant for Russian sturgeon. Only those peaks for which differences were determined to be significant (Student's t-test, $p < 0.05$, see Materials and Methods section in the main manuscript for details) were included. Note that the two difference spectra are displayed using the same Y-axis scale. The type of hydrogen (e.g., methyl) responsible for producing each peak is indicated parenthetically using underline (e.g., $C\underline{H}_3$). Key: Arg, arginine; Asp, aspartate; CHO, cholesterol; Cys, cysteine; Gln, glutamine; Glu, glutamate; Gly, glycine; His, histidine; Ile, isoleucine; Leu, leucine; L.L., lipoprotein lipids; Lys, lysine; Met, methionine; Phe, phenylalanine; Ser, serine; Thr, threonine; Trp, tryptophan; Tyr, tyrosine; Val, valine.

Supplementary Figure S6. The ^{1}H NMR spectrum (prior to the normalization and residual water / solvent peak removal steps that are described in the Material and Methods section of the main manuscript) of epidermal mucus from a representative (a) male and (b) female Russian sturgeon. The key that associates the spectrum-peak labels (i.e., the peak numbers) with the metabolites that give rise to the peaks is in Supplementary Table S3.

Supplementary Figure S7. The ^{1}H NMR spectrum (prior to the normalization and residual water / solvent peak removal steps that are described in the Material and Methods section of the main manuscript) of epidermal mucus from a representative (a) male and (b) female Siberian sturgeon. The key that associates the spectrum-peak labels (i.e., the peak numbers) with the metabolites that give rise to the peaks is in Supplementary Table S3.

Supplementary Figure S8. Average ^{1}H NMR difference spectra that compare the relative abundances of endogenous metabolites measured in the epidermal mucus of: (a) male versus female Russian sturgeon, and (b) male versus female Siberian sturgeon. Positive-going peaks represent metabolites that are relatively more abundant in females, while negative-going peaks represent those that are relatively more abundant in males. Only those peaks for which differences were determined to be significant (Student's t-test, $p < 0.05$, see Materials and Methods section in the main manuscript for details) were included. Note that the two difference spectra are displayed using the same Y-axis scale, and that the negative-going peak for betaine (3.26 ppm) in the bottom spectrum is off scale. Key: RUS, Russian sturgeon; SIB, Siberian sturgeon; Asp, aspartate; AXP, adenosine phosphate(s); Glu, glutamate; Phe, phenylalanine; Thr, threonine; UDP, uridine diphosphate; Val, valine.

Supplementary Figure S9. Average ^{1}H NMR difference spectra that compare the relative abundances of endogenous metabolites measured in the epidermal mucus of Russian sturgeon versus Siberian sturgeon for (a) males of both species and (b) females of both species. Positive-going peaks

represent metabolites that are relatively more abundant for Siberian sturgeon, while negative-going peaks represent those that are relatively more abundant for Russian sturgeon. Only those peaks for which differences were determined to be significant (Student's t-test, $p < 0.05$, see Materials and Methods section in the main manuscript for details) were included. Note that the two difference spectra are displayed using the same Y-axis scale. Key: Arg, arginine; Asn, asparagine; Asp, aspartate; AXP, adenosine phosphate(s); Gln, glutamine; Glu, glutamate; Thr, threonine; UDP, uridine diphosphate.

Supplementary Figure S10. Total intensity difference values for estimating dissimilarity between Russian sturgeon and Siberian sturgeon metabolomes (mean ± standard error) for the two sexes and two sample types, as measured using ^{1}H NMR spectra (see Materials and Method section in the main manuscript for details). Bars with different letters indicate significant differences based on ANOVA with posthoc Tukey's test, $p < 0.05$. The number (N) of individuals included in the comparison is listed under each bar. Key: RUS, Russian sturgeon; SIB, Siberian sturgeon.

SUPPLEMENTARY FIGURES –

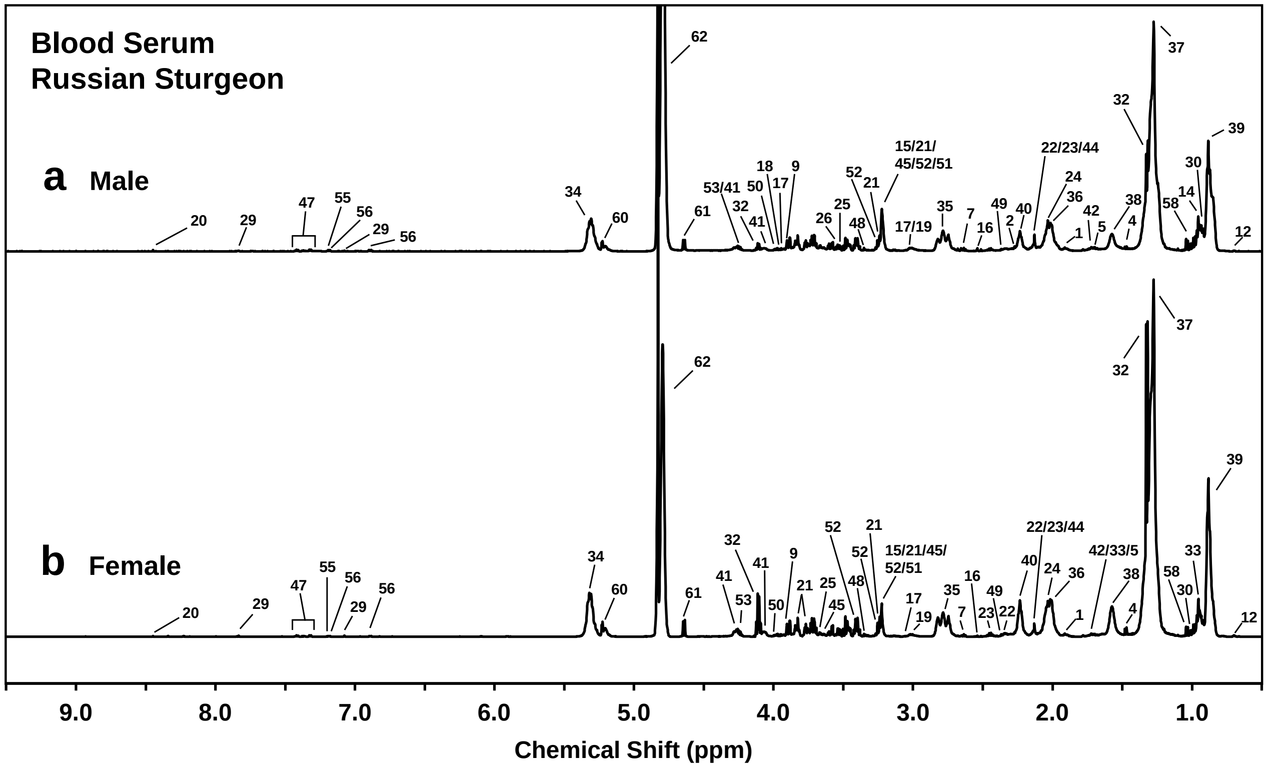

Supplementary Figure S1

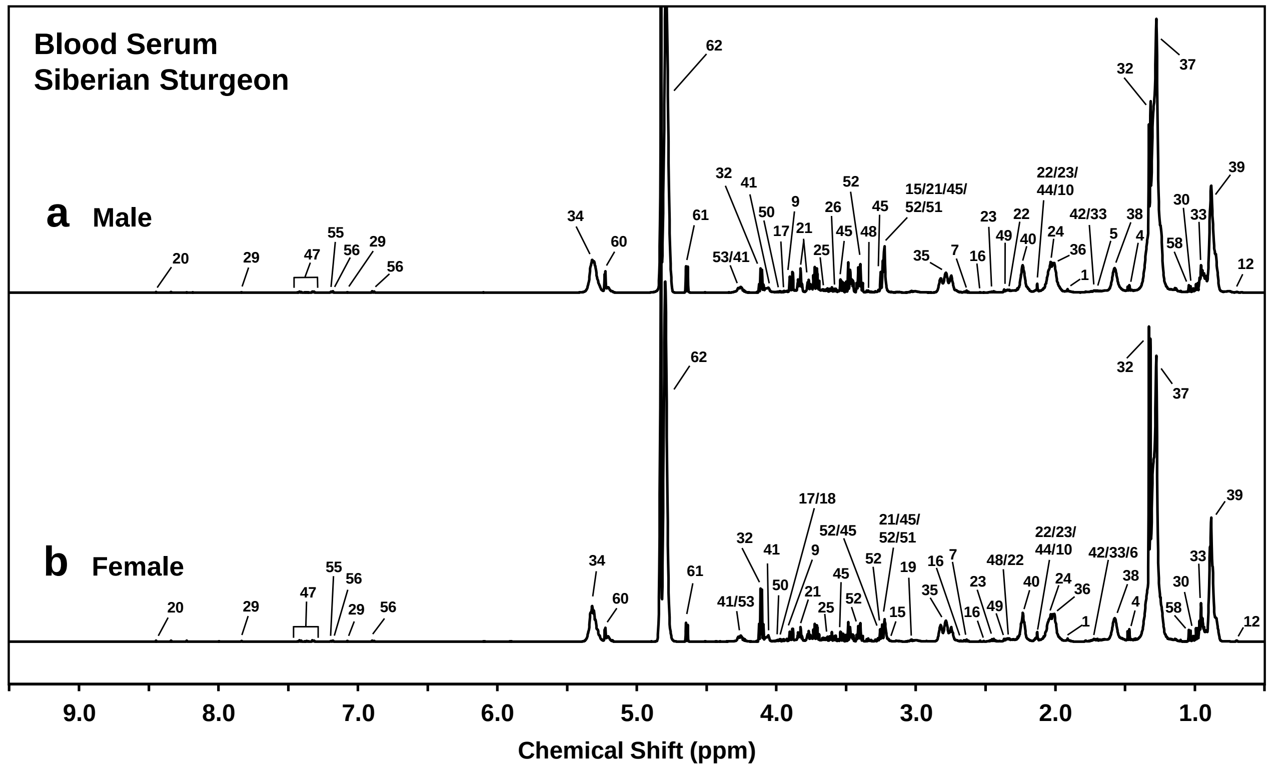

Supplementary Figure S2

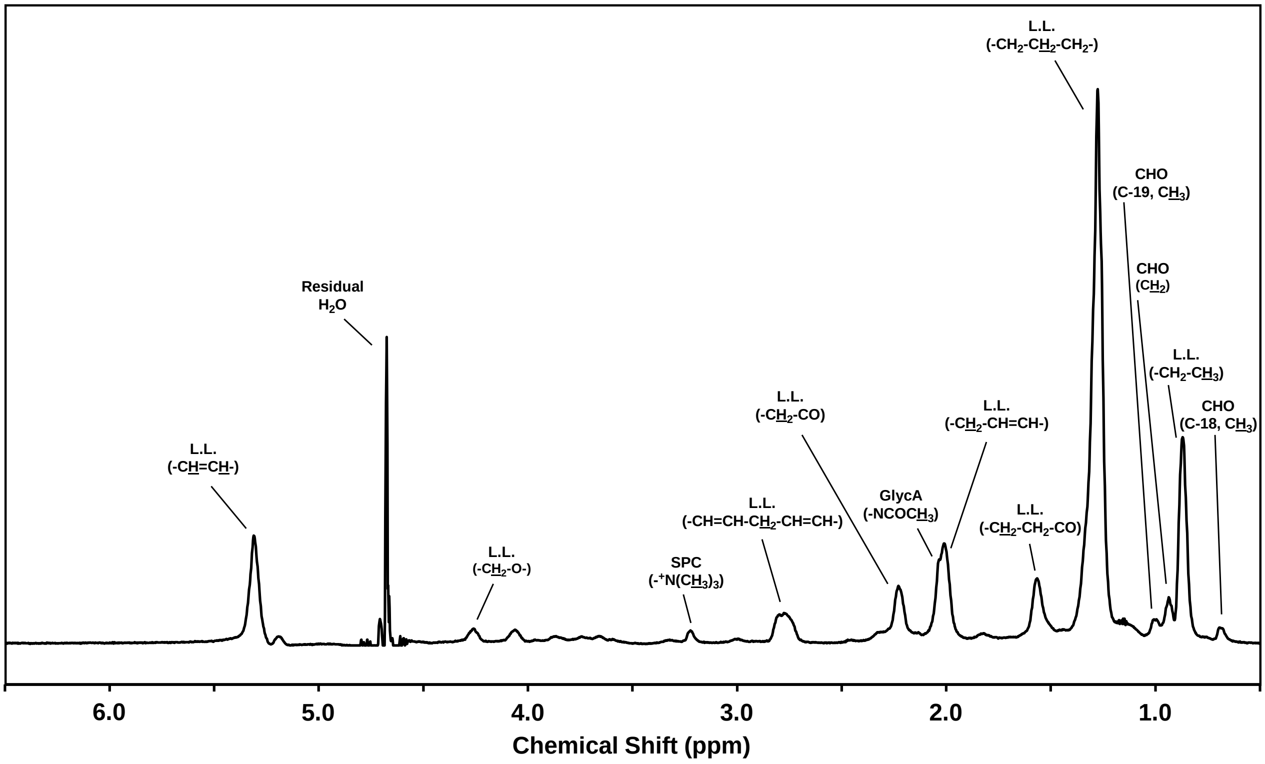

Supplementary Figure S3

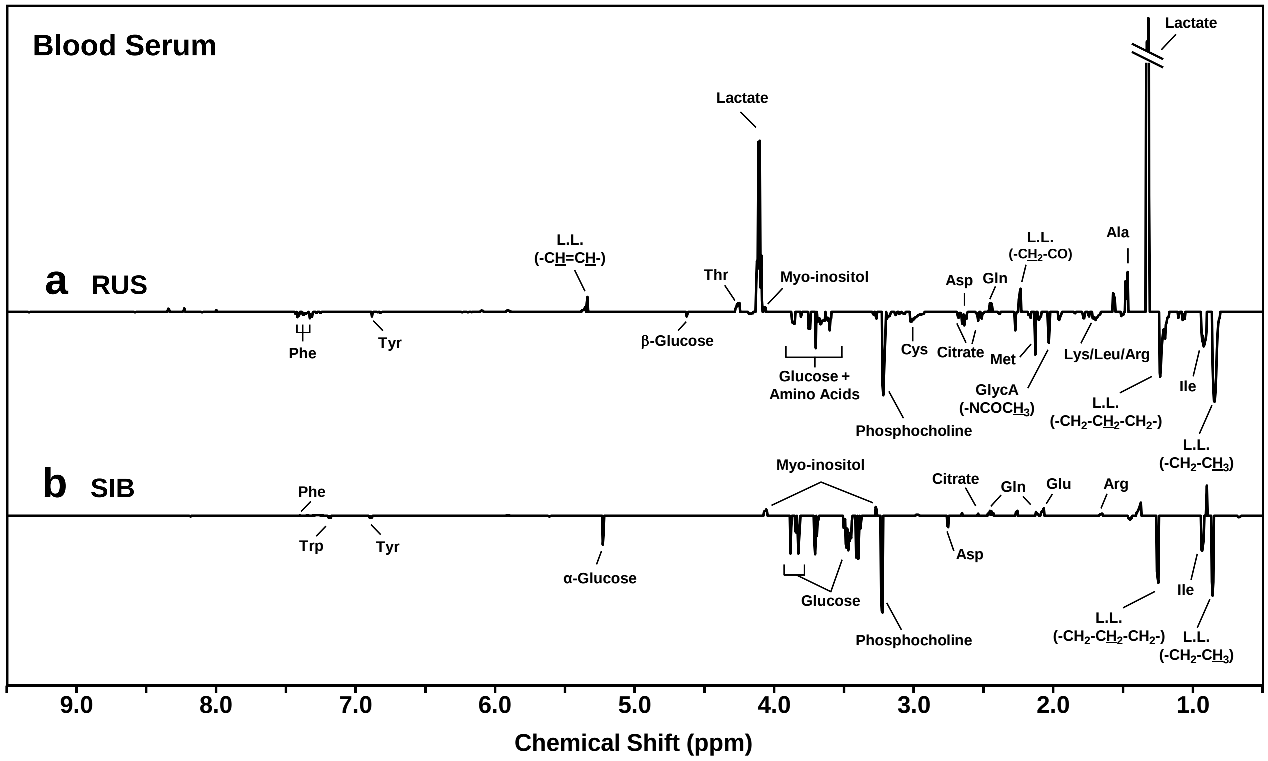

Supplementary Figure S4

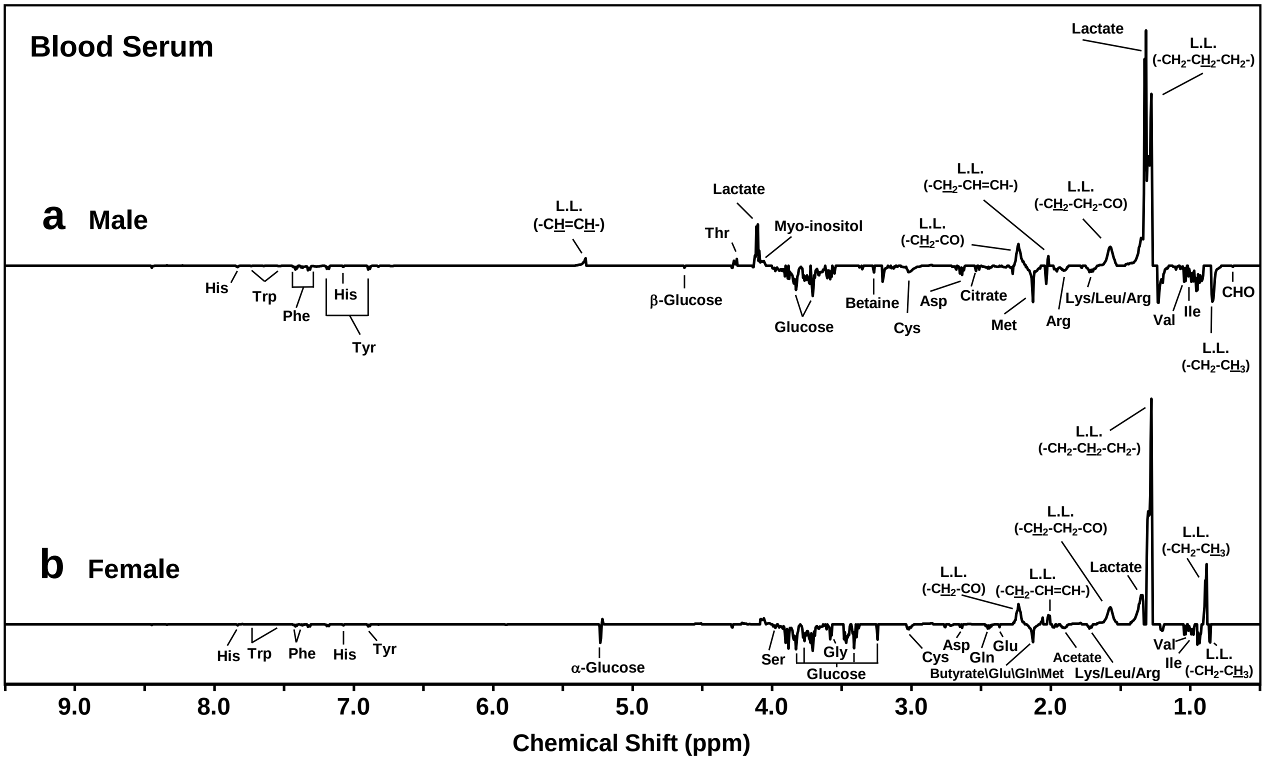

Supplementary Figure S5

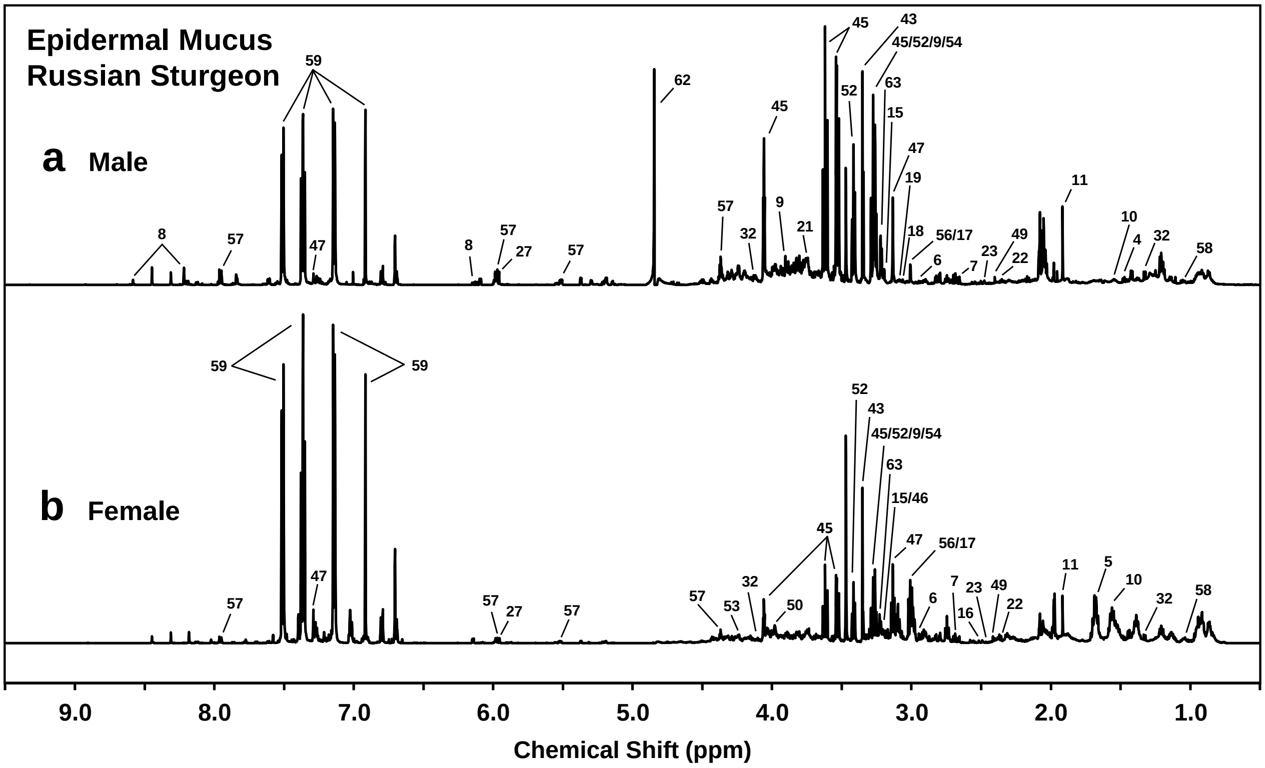

Supplementary Figure S6

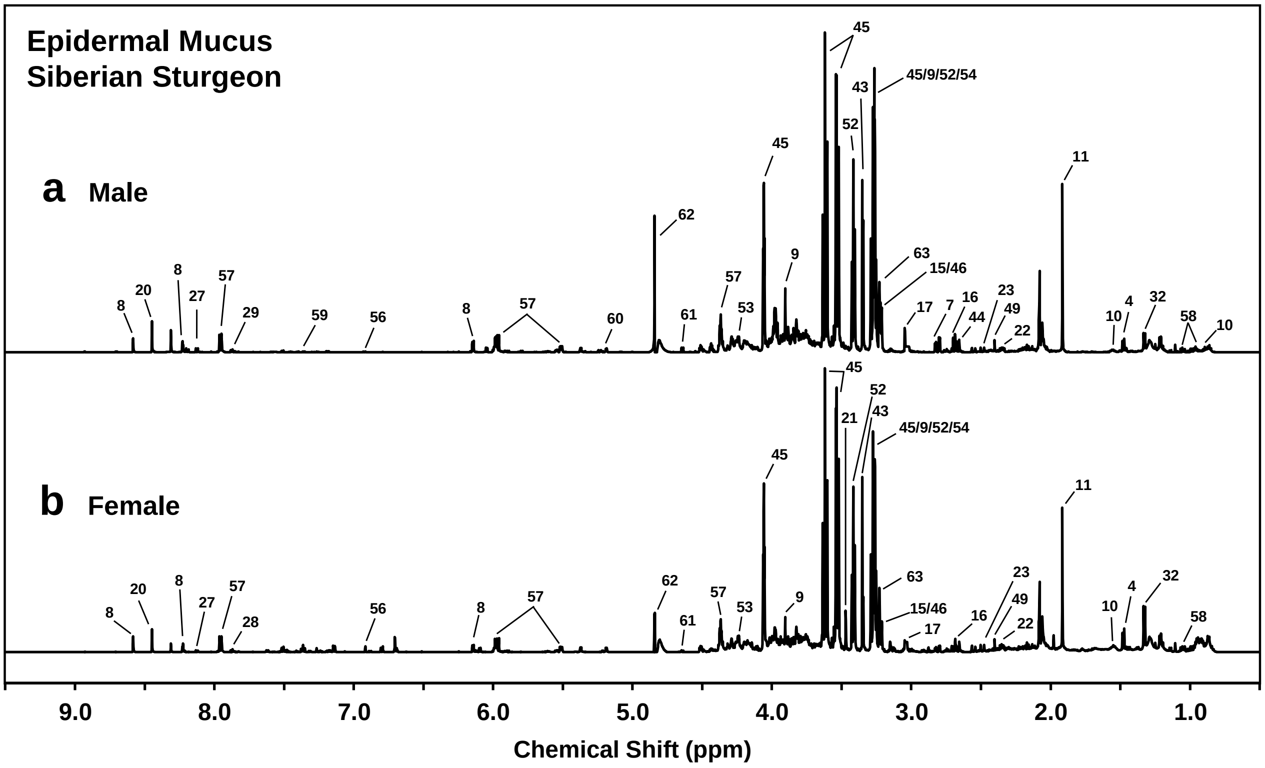

Supplementary Figure S7

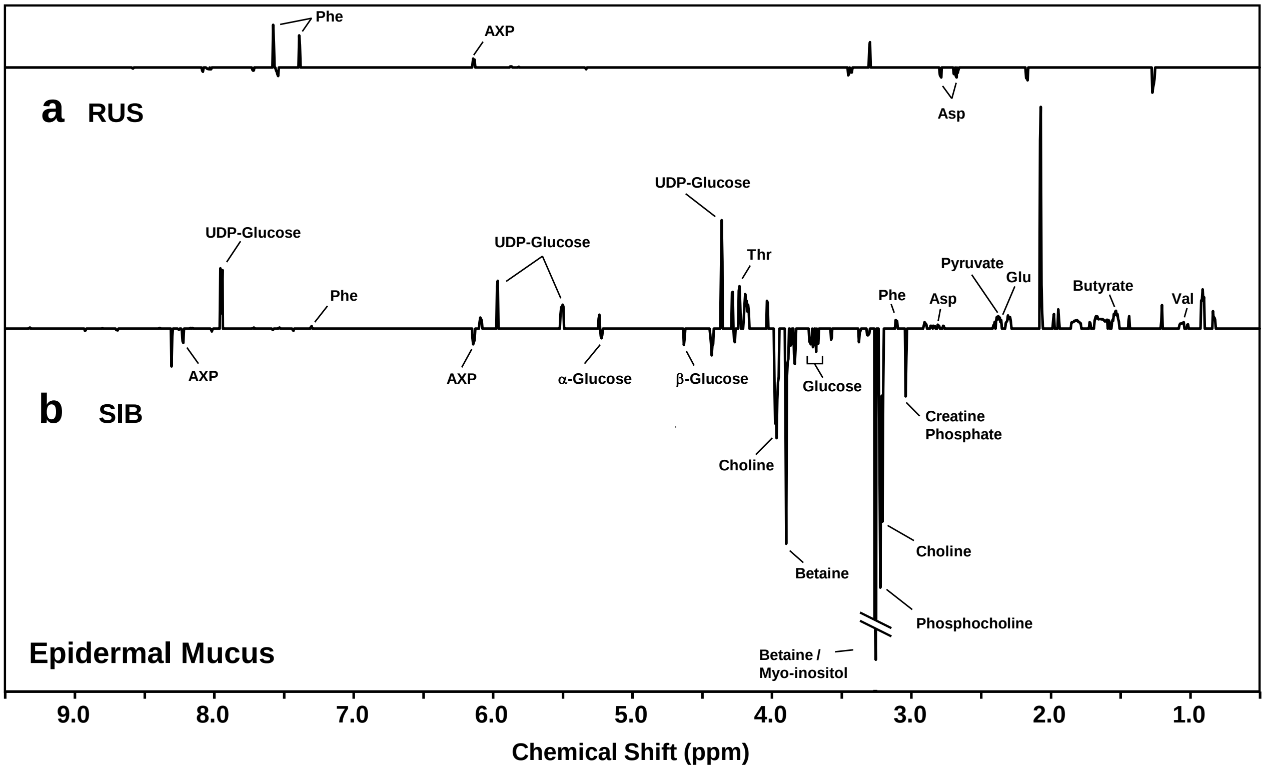

Supplementary Figure S8

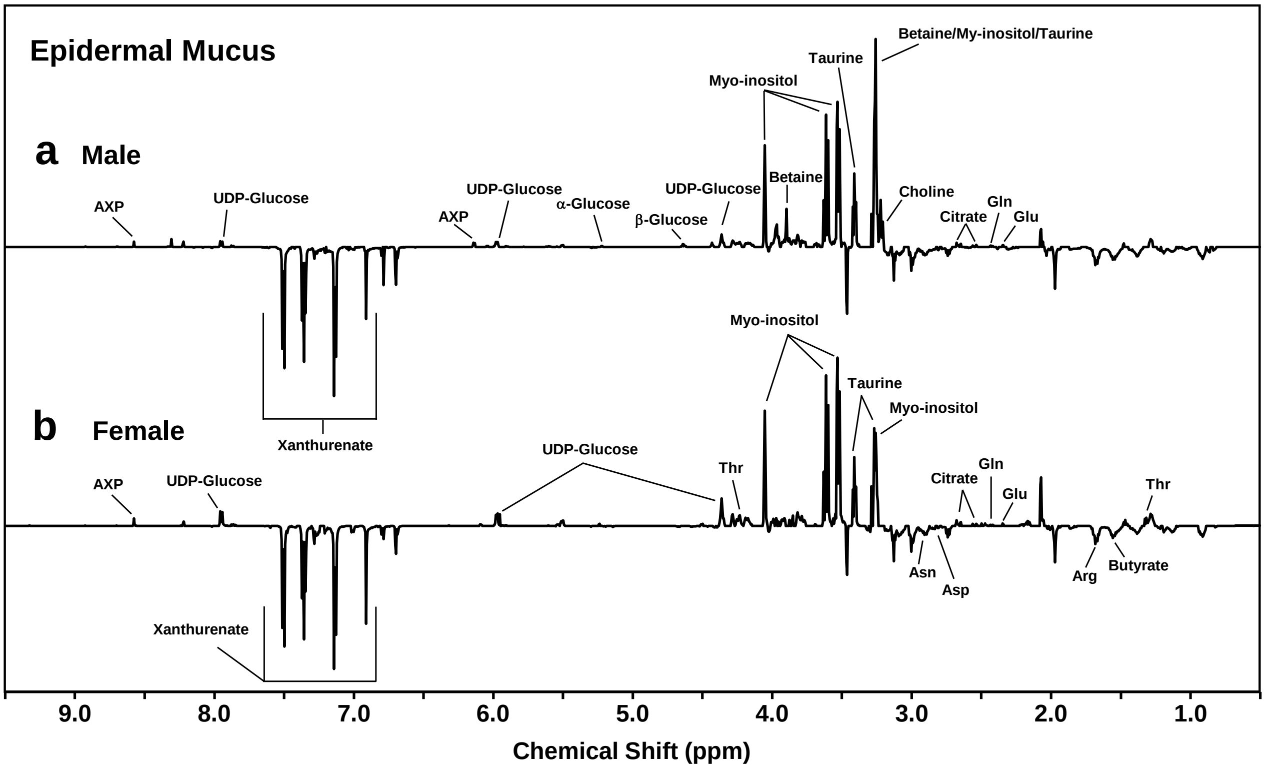

Supplementary Figure S9

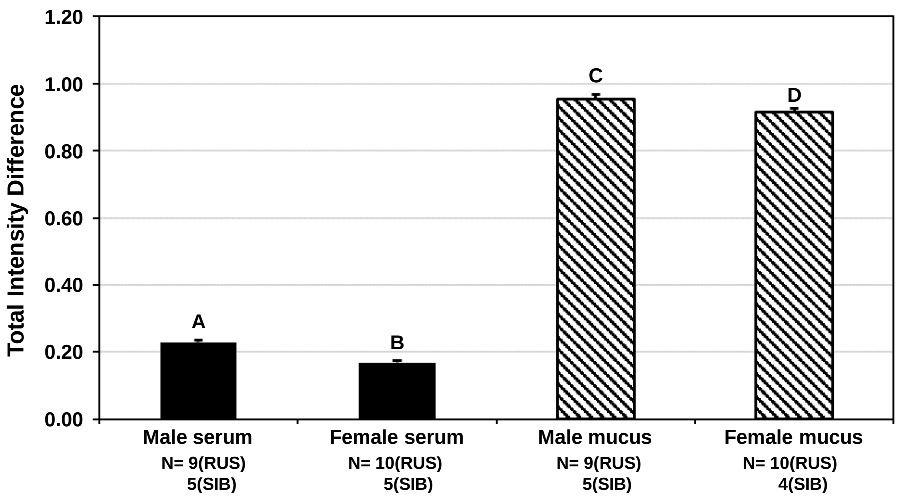

Supplementary Figure S10
